# Prognostic Significance of *CDKL2* in Non-Small Cell Lung Cancer: A Favorable Association in Lung Adenocarcinoma

**DOI:** 10.3390/medicina62091638

**Published:** 2026-08-27

**Authors:** Minji Song, Yunha Lee, Jun-Chae Lee, An-Na Bae, Jae-Ho Lee

**Affiliations:** 1 Medical Course, School of Medicine, Keimyung University, Daegu 42601, Republic of Korea; crazyfox2000@naver.com (M.S.);; 2 Department of Molecular Biology, Pusan National University, Busan 46241, Republic of Korea; 3 Department of Anatomy, School of Medicine, Keimyung University, Daegu 42601, Republic of Korea

**Keywords:** *CDKL2*, non-small cell lung cancer, lung adenocarcinoma, lung squamous cell carcinoma, TCGA, prognosis, single-cell RNA sequencing, tumor microenvironment

## Abstract

*Background and Objectives:* Non-small cell lung cancer (NSCLC) comprises biologically distinct histologic subtypes, including lung adenocarcinoma (LUAD) and lung squamous cell carcinoma (LUSC). We evaluated whether the prognostic association of cyclin-dependent kinase-like 2 (*CDKL2*) differs by histology. *Materials and Methods*: TCGA *CDKL2* expression was linked to harmonized clinical data. The primary analysis used a pooled multivariable Cox model with continuous standardized *CDKL2* expression, histology, and a *CDKL2* × histology interaction, adjusted for age, sex, pathologic stage, and smoking. Additional analyses assessed model assumptions, tumor purity, tumor-versus-normal expression, KEGG pathways, the tumor microenvironment, and single-cell RNA sequencing. External evaluation used GSE30219. *Results:* The primary TCGA cohort included 943 patients (471 LUAD, 472 LUSC; 375 deaths). Higher *CDKL2* expression was associated with lower mortality in LUAD (HR per 1 pooled SD = 0.721, 95% CI 0.603–0.862, *p* < 0.001) but higher mortality in LUSC (HR = 1.237, 95% CI 1.011–1.515, *p* = 0.039), with a significant interaction (HR = 1.717, *p* < 0.001) persisting after tumor-purity adjustment. *CDKL2*-low tumors were enriched for cell-cycle, DNA-replication, proteasome, and DNA-repair programs. Single-cell analyses showed predominant epithelial detection and greater malignant-cell *CDKL2* detection in LUAD. In GSE30219, the favorable LUAD association was supported in a median-split-adjusted analysis of 207073_at (HR = 0.473, 95% CI 0.250–0.897, *p* = 0.022), whereas the corresponding continuous estimate was directionally favorable but nonsignificant; 236331_at was nonsignificant in both models; neither probe supported the adverse LUSC association or histology interaction. *Conclusions:* Higher CDKL2 expression showed a favorable prognostic association in LUAD, with consistent TCGA sensitivity analyses and limited but suggestive independent-cohort support. The adverse LUSC association remains preliminary, and *CDKL2* should be regarded as a candidate prognostically associated marker rather than an established clinical biomarker.

## 1. Introduction

Lung cancer remains one of the leading causes of cancer-related mortality worldwide, and non-small cell lung cancer (NSCLC) accounts for the majority of lung cancer diagnoses [1,2]. Lung adenocarcinoma (LUAD) and lung squamous cell carcinoma (LUSC) are the two most common NSCLC histologies and differ substantially in molecular architecture, clinical characteristics, and therapeutic vulnerabilities [2]. Despite major advances in targeted therapy and immunotherapy, outcomes remain heterogeneous, supporting continued investigation of prognostically informative molecular features [2].

LUAD and LUSC should not be treated as a single molecular entity. Comprehensive genomic and transcriptomic characterization has demonstrated distinct molecular contexts across the two histologies. LUAD is frequently characterized by receptor-tyrosine-kinase/RAS-pathway alterations involving EGFR, KRAS, BRAF, and ALK-related events, whereas LUSC commonly shows TP53 disruption together with recurrent alterations and lineage-associated programs involving CDKN2A, PI3K-pathway genes, oxidative-stress pathways, and squamous-lineage regulators [3,4]. These differences provide a biological rationale for formally testing whether the prognostic association of a candidate gene differs by histology, rather than assuming such a difference based only on separate subgroup analyses [3,4].

Cyclin-dependent kinase-like 2 (*CDKL2*) belongs to the CDKL family of serine/threonine kinases [5,6]. CDKL family members share structural features with cyclin-dependent and mitogen-activated protein kinases but have evolved distinct regulatory characteristics [5,6]. Experimental work in breast cancer has linked *CDKL2* to epithelial-mesenchymal transition, migration, and tumor progression [7]. In contrast, the clinical relevance of *CDKL2* in lung cancer remains incompletely characterized, and evidence for histology-specific prognostic associations has been limited.

The present study therefore re-evaluated *CDKL2* in LUAD and LUSC using participant-level TCGA expression and harmonized clinical data, together with a continuous-expression survival model. The primary objective was to test the *CDKL2* × histology interaction in a pooled multivariable Cox model. Secondary and sensitivity analyses evaluated the robustness of the survival associations, tumor-microenvironmental and transcriptomic correlates of *CDKL2*, tumor-versus-normal expression, and the cellular distribution of *CDKL2* using single-cell data. The prognostic findings were further evaluated in the independent GSE30219 cohort, with additional sensitivity analyses to assess the consistency of the external findings.

## 2. Materials and Methods

### 2.1. Study Design, TCGA Data Sources, and Cohort Reconstruction

*CDKL2* mRNA expression data for TCGA-LUAD and TCGA-LUSC were obtained from OncoLnc on 13 August 2026 [8]. OncoLnc provides TCGA Tier 3 RNASeqV2 mRNA expression derived from rsem.genes.normalized_results files; the downloaded *CDKL2* values were therefore treated as normalized RSEM expression [8]. All TCGA-LUAD and TCGA-LUSC participants with available OncoLnc *CDKL2* expression data and corresponding participant-level clinical identifiers were eligible for cohort reconstruction. Expression data were available for 492 LUAD and 488 LUSC participants.

Expression records were matched by TCGA participant barcode to harmonized clinical data. Overall survival (OS), progression-free interval (PFI), age, sex, and pathologic stage were obtained from the TCGA Pan-Cancer Clinical Data Resource (TCGA-CDR; TCGA-CDR-Supplemental TableS1.xlsx; accessed 13 August 2026), whereas smoking history was obtained from the PanCanAtlas clinical follow-up dataset (clinical_PANCAN_patient_with_followup.tsv; accessed 13 August 2026) [9]. OS was defined according to the harmonized TCGA-CDR endpoint, with death from any cause considered an event and surviving participants censored at the last available follow-up. Participant barcodes were used as unique identifiers, and one record per participant was retained. The reconstructed master cohort contained 980 unique participants (492 LUAD and 488 LUSC). Missing clinical covariates were not imputed.

The primary multivariable survival analysis used complete cases for OS, *CDKL2* expression, age, sex, pathologic stage, and smoking status. Thirty-seven participants were excluded: 25 for missing smoking history only, 11 for missing pathologic stage only, and one for both variables. The primary cohort therefore comprised 943 participants (471 LUAD and 472 LUSC), including 375 deaths (168 LUAD and 207 LUSC). Patient selection is summarized in Supplementary Table S4.

### 2.2. CDKL2 Expression Processing and Grouping

For the primary TCGA survival analysis, normalized RSEM *CDKL2* expression was transformed as log2(expression + 1). The transformed values were standardized across the pooled LUAD/LUSC complete-case cohort, so the primary hazard ratios represent the change in mortality hazard per one pooled standard-deviation (SD) increase in *CDKL2* expression.

*CDKL2* was retained as a continuous variable for primary inferential analyses. For secondary Kaplan–Meier visualization and exploratory clinicopathological analyses, participants were classified as *CDKL2*-high or *CDKL2*-low according to the median expression within each histological subtype. The median cutoff was outcome-independent; however, because dichotomization discards continuous information and creates cohort-specific thresholds, these grouped analyses were considered secondary and were not interpreted as clinically established cutoffs.

### 2.3. TCGA Tumor–Normal Expression Analysis

To address potential confounding introduced by combining TCGA and GTEx normal tissues, a TCGA-only tumor–normal sensitivity analysis was performed using harmonized RNA-seq data retrieved from the Genomic Data Commons with TCGAbiolinks [10]. Primary-tumor and solid-tissue–normal samples from TCGA-LUAD and TCGA-LUSC were analyzed using the available tpm_unstrand TPM assay and transformed as log2(TPM + 1). When multiple RNA-seq aliquots of the same sample type were available for a participant, transformed expression values were averaged to obtain one participant-level value per sample type. The participant-level dataset included primary-tumor specimens from 517 LUAD patients and solid-tissue–normal specimens from 59 LUAD patients, and primary-tumor specimens from 501 LUSC patients and solid-tissue–normal specimens from 51 LUSC patients. Of these, 58 LUAD and 51 LUSC patients had matched tumor–normal specimens. Because most solid-tissue–normal specimens were matched to tumor specimens from the same participants, tumor–normal differences were formally evaluated using paired Wilcoxon signed-rank tests among the 58 matched LUAD and 51 matched LUSC participants. False-discovery-rate (FDR) adjustment across the two histology-specific paired comparisons was performed using the Benjamini–Hochberg method. GEPIA2 (accessed 15 August 2026) was retained only as a complementary pan-cancer expression resource because its normal reference incorporates both TCGA and GTEx tissues [11]. Accordingly, GEPIA2 was not used as the primary tumor-versus-normal comparison.

### 2.4. Clinicopathological Association Analysis

Clinicopathological associations were evaluated separately in the full expression-available LUAD and LUSC cohorts using histology-specific median *CDKL2* groups. Age was categorized as ≤65 versus >65 years, smoking as ever versus never smoking, and pathologic stage as stage I, stage II, or stage III/IV. Sex was analyzed as recorded in TCGA.

Categorical data are reported as n (%), with percentages calculated among non-missing observations within each *CDKL2* group. The number of missing observations is shown for every variable. Pearson’s chi-square test was used for comparisons of categorical variables. After regrouping pathologic stage and omitting sparse TNM subcategories from the final descriptive tables, all Table 1 and Table 2 comparisons satisfied Pearson chi-square assumptions. These analyses were considered exploratory; no multiplicity correction was applied to the clinicopathological comparisons.

### 2.5. TCGA Survival Analysis

OS, as defined by the TCGA-CDR, was the primary survival endpoint [9]. The primary inferential analysis was a pooled multivariable Cox proportional-hazards model: Surv(OS time, OS event) ~ standardized *CDKL2* × histology + age + sex + pathologic stage + smoking. LUAD was the reference histology. Age was entered continuously. Pathologic stage was modeled categorically as stage I (reference), stage II, and stage III/IV. TCGA-CDR stage entries classified as discrepant or unavailable were treated as missing. Smoking was categorized as ever versus never smoking using the PanCanAtlas tobacco-smoking-history variable. Current smokers and former or reformed smokers were classified as ever smokers, whereas lifelong nonsmokers were classified as never smokers. Smoking entries classified as unavailable, unknown, or discrepant were treated as missing. The *CDKL2* main coefficient represents the LUAD-specific effect, whereas the LUSC-specific *CDKL2* effect was obtained from the linear combination of the *CDKL2* main coefficient and the *CDKL2* × LUSC interaction coefficient.

For secondary graphical presentation, Kaplan–Meier curves were generated separately for LUAD and LUSC using the histology-specific median *CDKL2* expression value. Groups were compared using the log-rank test, and numbers at risk and censoring marks were displayed. These median-based analyses were considered secondary to the continuous-expression Cox analysis.

The proportional-hazards assumption was assessed using Schoenfeld residuals. Because sex and pathologic stage showed evidence of nonproportionality in the primary model, a sensitivity Cox model stratified by sex and pathologic stage was fitted. Potential nonlinearity was assessed separately in LUAD and LUSC using restricted cubic splines with three knots. A further sensitivity analysis divided *CDKL2* into histology-specific quartiles, with Q1 as the reference and an ordinal trend test across quartiles.

Progression-free interval (PFI) was evaluated as an exploratory secondary endpoint using the TCGA-CDR [9]. PFI was defined as the interval from diagnosis to the first occurrence of a new tumor event, including disease progression, locoregional recurrence, distant metastasis, a new primary tumor, or death with tumor; participants without a PFI event were censored according to the TCGA-CDR definition. PFI data were available for all 492 LUAD and 488 LUSC participants, with 200 and 144 PFI events, respectively. Median follow-up was estimated using the reverse Kaplan–Meier method and was 761 days (95% CI, 690–888) for LUAD and 833 days (95% CI, 741–960) for LUSC.

The main inferential PFI analysis used a pooled multivariable Cox proportional-hazards model with continuous standardized *CDKL2* expression, histologic subtype, the *CDKL2* × histologic subtype interaction, age, sex, pathologic stage, and smoking status, using the same coding and pooled standardization approach as the primary OS model. PFI was available for all 980 participants; therefore, the 37 exclusions from the multivariable PFI model reflected missing adjustment covariates rather than missing PFI data. Application of the same complete-case criteria used for the primary OS model yielded 943 participants and 323 PFI events. The proportional-hazards assumption was evaluated using Schoenfeld residuals, with both term-specific and global tests examined. Because pathologic stage showed marked evidence of nonproportionality, a sensitivity Cox model stratified by pathologic stage was additionally fitted. Proportional-hazards diagnostics were reassessed in the stage-stratified model.

For secondary graphical presentation, Kaplan–Meier curves were generated separately for LUAD and LUSC using the histology-specific median *CDKL2* expression value, and groups were compared using the log-rank test. Numbers at risk and censoring marks were displayed. These median-based analyses were considered descriptive and secondary to the continuous-expression Cox analysis. PFI analyses were considered exploratory and were not externally validated.

### 2.6. External Evaluation Using the GSE30219 Dataset

The independent GSE30219 cohort was obtained from the NCBI Gene Expression Omnibus and originated from the lung cancer series reported by Rousseaux et al. [12]. GSE30219 was generated on the Affymetrix Human Genome U133 Plus 2.0 Array (GPL570). Processed expression values deposited in GEO were used; according to the GEO record, the data were normalized using the robust multi-array average (RMA) algorithm in GeneSpring, without baseline transformation. Histological subtype was assigned from the GEO histology annotation, with ADC classified as LUAD and SQC as LUSC. The expression cohort contained 85 LUAD and 61 LUSC tumors. Two LUAD cases with an OS time of 0 months were excluded, leaving 83 LUAD and 61 LUSC cases in the survival-analysis cohort. Treatment information was not available in a sufficiently harmonized form for inclusion as a covariate. Smoking history and a harmonized overall pathologic-stage variable were also unavailable.

Current hgu133plus2 annotation identified two CDKL2-mapped probe sets, 207073_at and 236331_at. Because neither probe set could be unambiguously prioritized on annotation grounds, external survival analyses were performed in parallel for both probe sets. Within each histology, patients were dichotomized at the histology-specific median expression value of each probe set for Kaplan–Meier analysis with the log-rank test and for multivariable Cox regression adjusted for age, sex, T stage, and N stage.

For each probe set, continuous expression was standardized across the pooled LUAD and LUSC survival cohort. A pooled multivariable Cox model included standardized CDKL2 expression, histologic subtype, and a CDKL2 × histology interaction term, with adjustment for age, sex, T stage, and N stage. From each probe-specific pooled model, the CDKL2 effect was estimated separately for LUAD and LUSC, together with the CDKL2 × histology interaction.

Because advanced T- and N-stage categories were sparse, sensitivity analyses were additionally performed using collapsed T stage (T1 versus T2–4) and N stage (N0 versus N1+). With this collapsed-stage coding, continuous and median-based Cox models were evaluated separately within LUAD and LUSC for both probe sets, and the pooled continuous CDKL2 × histology interaction model was repeated for each probe set. Proportional-hazards assumptions were assessed using Schoenfeld residuals for the pooled interaction models and the collapsed-stage median-based models. Model convergence and numerical stability were also assessed for the collapsed-stage median-based models.

### 2.7. KEGG Gene-Set Enrichment Analysis

A TCGA pathway analysis was performed in R using TCGAbiolinks, edgeR, limma, and clusterProfiler [10,13,14]. Within LUAD and LUSC separately, tumors were divided at the histology-specific median *CDKL2* expression. Gene-level statistics were generated using a limma-voom contrast defined as *CDKL2*-high minus *CDKL2*-low [13]. Genes were ranked by the resulting differential-expression statistic, and KEGG gene-set enrichment analysis was performed using clusterProfiler, following the general GSEA framework [14,15].

Positive normalized enrichment scores (NESs) therefore indicate enrichment toward *CDKL2*-high tumors, whereas negative NESs indicate enrichment toward *CDKL2*-low tumors. Pathways with FDR q < 0.05 were considered significant. The complete results for all 354 tested KEGG pathways in each histology, including pathway name, NES, nominal *p*-value, adjusted *p*-value, q-value, gene-set size, and leading-edge genes, are provided in Supplementary Table S3.

### 2.8. Exploratory Transcript Correlations and Tumor Microenvironment Analyses

Exploratory transcript-level correlations between *CDKL2* and selected histology-relevant genes were evaluated separately in LUAD and LUSC using TCGA RNA-seq data obtained from the Genomic Data Commons through TCGAbiolinks. Gene expression was quantified as transcripts per million (TPM) and transformed as log2(TPM + 1). When multiple primary-tumor RNA-seq aliquots were available for the same participant, transformed expression values were averaged at the participant level. The resulting expression data were matched to the reconstructed TCGA cohort by participant barcode, yielding 492 LUAD and 488 LUSC participants with complete expression and age data.

Genes were selected to represent histology-relevant oncogenic, tumor-suppressor, and lineage-associated molecular features described in prior genomic characterizations of LUAD and LUSC. The LUAD analysis included *EGFR*, *KRAS*, *TP53*, *ALK*, and *BRAF*, whereas the LUSC analysis included *TP53*, *CDKN2A*, *SOX2*, *PIK3CA*, and *NOTCH1*; age was additionally evaluated as an exploratory clinical correlate. Spearman rank correlation was used as the primary correlation measure to reduce sensitivity to distributional assumptions, with Pearson correlation additionally performed as a sensitivity analysis. Benjamini–Hochberg correction was applied separately across the six comparisons within each histologic subtype and correlation method. Gene–gene analyses evaluated mRNA-to-mRNA co-expression and were not interpreted as measures of somatic mutation, copy-number alteration, gene-fusion status, or pathway activation. Because these analyses were exploratory, they were not used to support the primary prognostic conclusions.

TIMER2.0 (accessed 15 August 2026) was used as a complementary resource for continuous survival and tumor-purity-adjusted partial Spearman correlation analyses [16]. Because TIMER2.0 is largely based on TCGA samples, it was considered a sensitivity resource rather than an independent validation cohort.

To assess potential confounding by tumor purity, ESTIMATE tumor-purity estimates from the TCGA tumor-purity dataset reported by Aran et al. were matched to the reconstructed TCGA cohort by participant barcode [17]. When multiple tumor samples were available for the same participant, a primary tumor sample was preferentially retained, with the 01A vial prioritized when available. Within each histologic subtype, the association between *CDKL2* expression and estimated tumor purity was evaluated using Spearman correlation. Tumor purity was then added to the primary pooled Cox model to assess whether adjustment for bulk-tumor cellular composition materially altered the *CDKL2* × histology association. ESTIMATE purity was available for 939 of the 943 participants in the primary complete-case survival cohort.

Precomputed ESTIMATE-derived ImmuneScore and StromalScore values for TCGA-LUAD and TCGA-LUSC were obtained from the ESTIMATE disease-centric resource (RNA-seqV2; accessed 16 August 2026) [18] and matched to the primary TCGA cohort by participant barcode.

To further characterize associations between *CDKL2* expression and the immune microenvironment, immune-cell infiltration was evaluated using the TIMER Gene module [19]. TIMER estimates the abundance of six major tumor-infiltrating immune-cell populations: B cells, CD8+ T cells, CD4+ T cells, macrophages, neutrophils, and dendritic cells. Analyses were performed separately for LUAD and LUSC, and associations between *CDKL2* expression and estimated immune-cell abundance were assessed using tumor-purity-adjusted partial Spearman correlations. Benjamini–Hochberg correction was applied across the 12 histology–cell-type comparisons. These analyses were considered exploratory and were interpreted as associations with estimated immune-cell abundance rather than evidence of direct cellular expression or causal immune regulation.

### 2.9. Exploratory Single-Cell RNA-Sequencing Analysis

Publicly available single-cell RNA-sequencing data from the Lung Cancer Atlas (LuCA) core atlas were used to characterize the cellular distribution of *CDKL2* expression [20]. The LuCA core atlas H5AD dataset was downloaded from CZ CELLxGENE on 15 August 2026. Analyses were restricted to cells annotated as originating from primary LUAD or LUSC tumors. Existing LuCA cell-type annotations were retained without re-clustering or re-annotation. *CDKL2*-positive cells were defined as cells with detectable *CDKL2* counts (>0), and detection frequencies were summarized across the coarse cell-type annotations provided in the atlas. Epithelial annotations were additionally examined to determine whether *CDKL2* detection was restricted to annotated malignant cells.

To avoid treating individual cells as independent biological replicates and to reduce instability in donor-level detection estimates based on small cell numbers, malignant-cell analyses were summarized by donor and restricted to samples containing at least 50 annotated malignant cells. Sensitivity analyses were repeated using minimum thresholds of 100 and 200 malignant cells per donor. Because the integrated LuCA atlas combines multiple source studies, the overall LUAD–LUSC donor-level comparison was treated as descriptive.

To reduce potential confounding by source-study differences, a within-study analysis was performed in the Wu_Zhou_2021 cohort [21], which contained both LUAD and LUSC primary tumors profiled within the same study using the same single-cell RNA-sequencing platform. Differences in the proportion of *CDKL2*-positive malignant cells were assessed using the Wilcoxon rank-sum test. In parallel, atlas-provided malignant-cell count values were aggregated within each donor to generate pseudobulk profiles, and differential *CDKL2* expression between LUAD and LUSC was assessed using DESeq2 with histology as the explanatory variable.

### 2.10. Statistical Software

Statistical analyses were performed in R versions (versions 4.5.3 and 4.6.1; R Foundation for Statistical Computing, Vienna, Austria). Key packages included survival, rms, survminer, ggplot2, TCGAbiolinks, GEOquery, edgeR, limma, clusterProfiler, readr, and dplyr. In the R 4.5.3 environment, key package versions included survival 3.8-6, rms 8.1-1, survminer 0.5.2, TCGAbiolinks 2.38.0, GEOquery 2.78.0, edgeR 4.8.2, limma 3.66.0, and clusterProfiler 4.18.4. In the R 4.6.1 environment, key package versions included survival 3.8.9, survminer 0.5.2, ggplot2 4.0.3, TCGAbiolinks 2.40.0, readr 2.2.0, and dplyr 1.2.1. Pearson and Spearman correlations and Benjamini–Hochberg adjustments were calculated using base R statistical functions. Single-cell analyses were performed in R version 4.6.1 using zellkonverter 1.22.0, SingleCellExperiment 1.34.0, scuttle 1.22.0, and DESeq2 1.52.0. All reported *p*-values were two-sided unless otherwise stated.

## 3. Results

### 3.1. CDKL2 Expression in TCGA Tumor and Normal Lung Tissues

**Figure 1:**
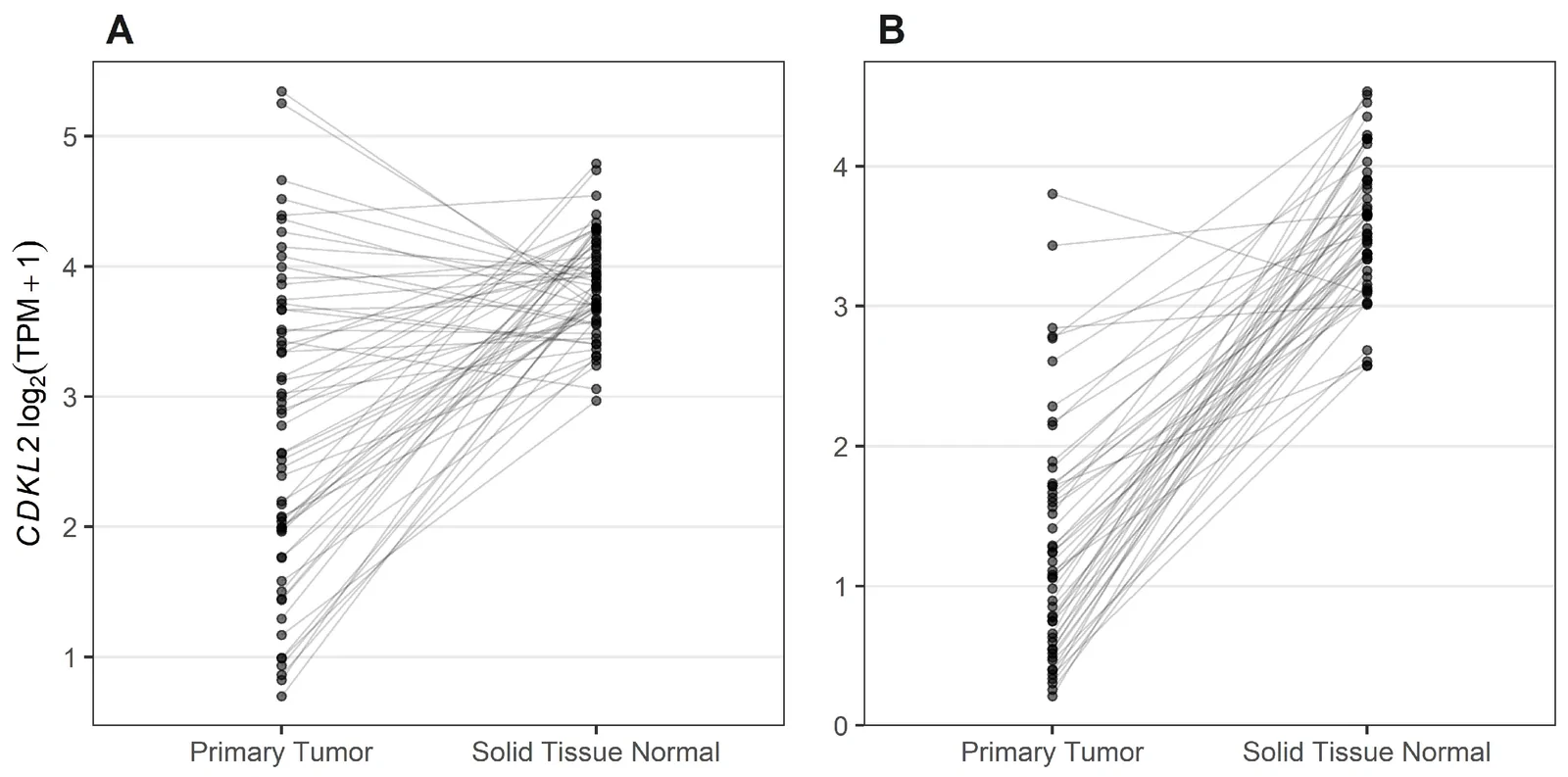
Paired TCGA tumor–normal comparison of *CDKL2* expression. Each line connects primary-tumor and solid-tissue–normal specimens obtained from the same participant. (**A**) LUAD: 58 matched tumor–normal pairs; *CDKL2* expression was significantly lower in tumor tissue (paired Wilcoxon signed-rank *p* = 7.83 × 10^−7^). (**B**) LUSC: 51 matched tumor–normal pairs; *CDKL2* expression was likewise significantly lower in tumor tissue (paired Wilcoxon signed-rank *p* = 6.34 × 10^−10^). Both histology-specific paired comparisons remained significant after Benjamini–Hochberg correction (FDR q = 7.83 × 10^−7^ for LUAD and 1.27 × 10^−9^ for LUSC). The reported paired Wilcoxon *p*-values were calculated exclusively from the matched subsets shown in the figure.

In the TCGA-only GDC analysis, participant-level median log2(TPM + 1) *CDKL2* expression in LUAD was 2.957 among 517 participants contributing primary-tumor specimens and 3.849 among 59 participants contributing solid-tissue–normal specimens. Formal tumor–normal inference was restricted to participants with matched specimens. Among the 58 matched LUAD participants, *CDKL2* expression was significantly lower in tumor tissue (paired Wilcoxon *p* = 7.83 × 10^−7^; Figure 1A). In LUSC, median expression was 1.161 among 501 participants contributing primary-tumor specimens and 3.519 among 51 participants contributing solid-tissue–normal specimens. The paired comparison among the 51 participants with matched specimens likewise showed lower *CDKL2* expression in tumor tissue (paired Wilcoxon *p* = 6.34 × 10^−10^; Figure 1B). Both histology-specific paired comparisons remained significant after Benjamini–Hochberg adjustment (FDR q = 7.83 × 10^−7^ for LUAD and 1.27 × 10^−9^ for LUSC). Complementary GEPIA2 analysis using the combined TCGA/GTEx normal reference showed lower expression in LUSC but not a significant LUAD difference, indicating that the apparent tumor–normal contrast is sensitive to the normal-reference framework [11].

### 3.2. Clinicopathological Associations

Among 492 LUAD participants, the median split yielded 246 *CDKL2*-low and 246 *CDKL2*-high tumors. High *CDKL2* expression was associated with a lower prevalence of ever-smoking history (80.1% versus 90.7%, *p* = 0.001). Associations with age group (*p* = 0.176), sex (*p* = 0.057), and grouped pathologic stage (*p* = 0.323) were not statistically significant (Table 1).

Among 488 LUSC participants, 244 tumors were assigned to each *CDKL2* group. *CDKL2* expression was associated with grouped pathologic stage (*p* = 0.002): stage I disease accounted for 56.8% of evaluable *CDKL2*-high tumors versus 42.0% of *CDKL2*-low tumors. The association with sex was borderline (*p* = 0.050), whereas age group (*p* = 0.227) and smoking status (*p* = 0.346) were not significant (Table 2).

### 3.3. Primary TCGA Survival Analysis and Sensitivity Analyses

The primary pooled TCGA analysis included 943 complete cases (471 LUAD and 472 LUSC) with 375 deaths. In the multivariable Cox model, each one pooled-SD increase in *CDKL2* expression was associated with lower mortality in LUAD (HR = 0.721, 95% CI 0.603–0.862, *p* < 0.001) but higher mortality in LUSC (HR = 1.237, 95% CI 1.011–1.515, *p* = 0.039). The *CDKL2* × histology interaction was significant (HR = 1.717, 95% CI 1.313–2.246, *p* < 0.001), demonstrating statistical heterogeneity of the *CDKL2*-OS association between LUAD and LUSC in TCGA (Table 3).

**Figure 2:**
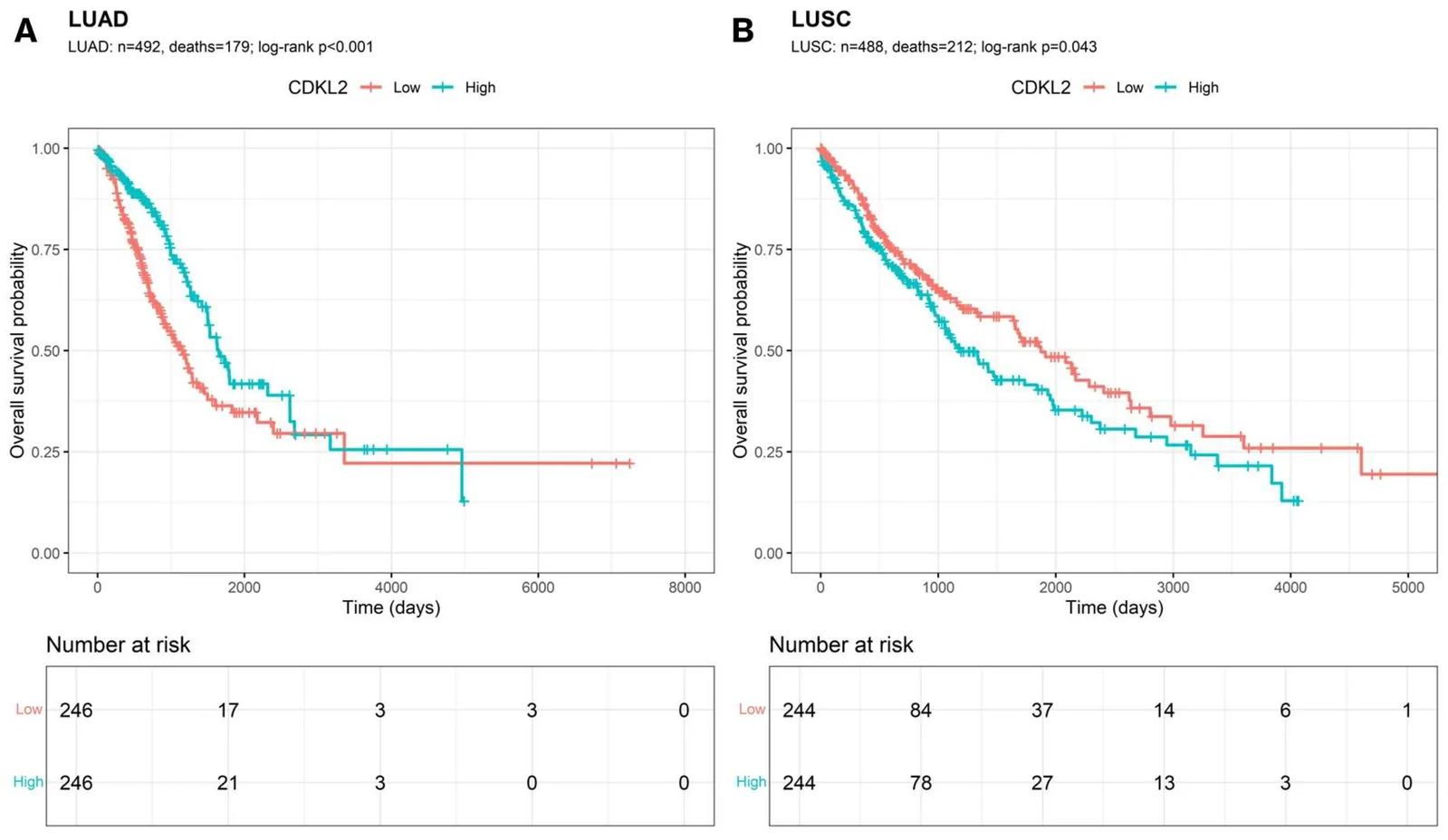
Kaplan–Meier analysis of overall survival according to *CDKL2* expression in TCGA-LUAD and TCGA-LUSC. Participants were divided at the median *CDKL2* expression value within each histology for visualization. (**A**) LUAD: n = 492, 179 deaths; high *CDKL2* expression was associated with longer OS (log-rank *p* = 0.00036). (**B**) LUSC: n = 488, 212 deaths; high *CDKL2* expression was associated with shorter OS (log-rank *p* = 0.0427). Numbers at risk and censoring marks are shown. Primary inferential analyses modeled *CDKL2* continuously.

Secondary median-based Kaplan–Meier analysis in the full expression-available cohorts showed the same directional pattern (Figure 2). In LUAD (n = 492; 179 deaths), the *CDKL2*-high group had 73 deaths among 246 participants versus 106 deaths among 246 *CDKL2*-low participants (log-rank *p* = 0.00036). In LUSC (n = 488; 212 deaths), there were 114 deaths among 244 *CDKL2*-high participants and 98 among 244 *CDKL2*-low participants (log-rank *p* = 0.0427).

Schoenfeld-residual testing identified nonproportionality for sex (*p* = 0.045) and pathologic stage (*p* = 0.010), with a significant global test (*p* = 0.012), whereas the *CDKL2* × histology interaction itself showed no evidence of nonproportionality (*p* = 0.962). In a sensitivity model stratified by sex and pathologic stage, the global proportional-hazards test was no longer significant (*p* = 0.378), and the interaction remained significant (HR = 1.764, 95% CI 1.350–2.305, *p* < 0.001) (Supplementary Table S5A,B).

Restricted cubic splines showed no evidence of nonlinearity in LUAD (*p* = 0.760) or LUSC (*p* = 0.991). Quartile analysis showed a graded favorable association in LUAD: compared with Q1, adjusted HRs were 0.877 for Q2, 0.557 for Q3, and 0.477 for Q4, with a significant trend (HR per quartile = 0.765, 95% CI 0.664–0.881, *p* < 0.001). In LUSC, HRs increased from 1.076 in Q2 to 1.300 in Q3 and 1.429 in Q4, although the trend narrowly missed conventional significance (HR per quartile = 1.135, 95% CI 0.998–1.292, *p* = 0.054) (Supplementary Table S5C,D).

PFI was evaluated as an exploratory secondary endpoint in all 492 LUAD and 488 LUSC participants, with 200 and 144 PFI events, respectively. In Kaplan–Meier analyses, the *CDKL2*-high group showed longer PFI than the *CDKL2*-low group in LUAD (median PFI, 1202 days [95% CI, 1018–2045] vs. 795 days [95% CI, 697–1433]; log-rank χ^2^ = 5.401, *p* = 0.020). In contrast, no significant difference was observed in LUSC (median PFI, 1990 days [95% CI, 1693–not estimable] vs. 2291 days [95% CI, 1656–not estimable]; log-rank χ^2^ = 0.058, *p* = 0.810) (Supplementary Figure S6).

In the continuous multivariable Cox analysis, higher *CDKL2* expression was associated with a lower risk of PFI events in LUAD (HR per 1 pooled-SD increase = 0.756, 95% CI 0.635–0.900, *p* = 0.002), whereas no significant association was observed in LUSC (HR = 1.138, 95% CI 0.891–1.454, *p* = 0.301). The *CDKL2* × histologic subtype interaction was significant (interaction HR = 1.506, 95% CI 1.117–2.029, *p* = 0.007), indicating statistical heterogeneity in the association between *CDKL2* expression and PFI across the two histologies.

The primary PFI Cox model showed evidence of nonproportionality for *CDKL2* expression (*p* = 0.010) and pathologic stage (*p* < 0.001), with a significant global test (*p* = 0.001); no evidence of nonproportionality was observed for the *CDKL2* × histologic subtype interaction (*p* = 0.329). In the stage-stratified sensitivity model, evidence of nonproportionality for CDKL2 was attenuated and was no longer statistically significant (*p* = 0.056); no evidence of nonproportionality was observed for the CDKL2 × histologic subtype interaction (*p* = 0.744), and the global proportional-hazards test was nonsignificant (*p* = 0.216). The interaction remained significant (HR = 1.475, 95% CI 1.094–1.989, *p* = 0.011). The favorable association in LUAD also remained significant (HR = 0.752, 95% CI 0.632–0.896, *p* = 0.001), whereas the association in LUSC remained nonsignificant (HR = 1.110, 95% CI 0.868–1.420, *p* = 0.406) (Supplementary Table S13A,B). Because the CDKL2-specific Schoenfeld result remained close to the conventional significance threshold after stage stratification, the constant PFI hazard-ratio estimates should be interpreted cautiously.

### 3.4. KEGG Pathway-Level Associations

**Figure 3:**
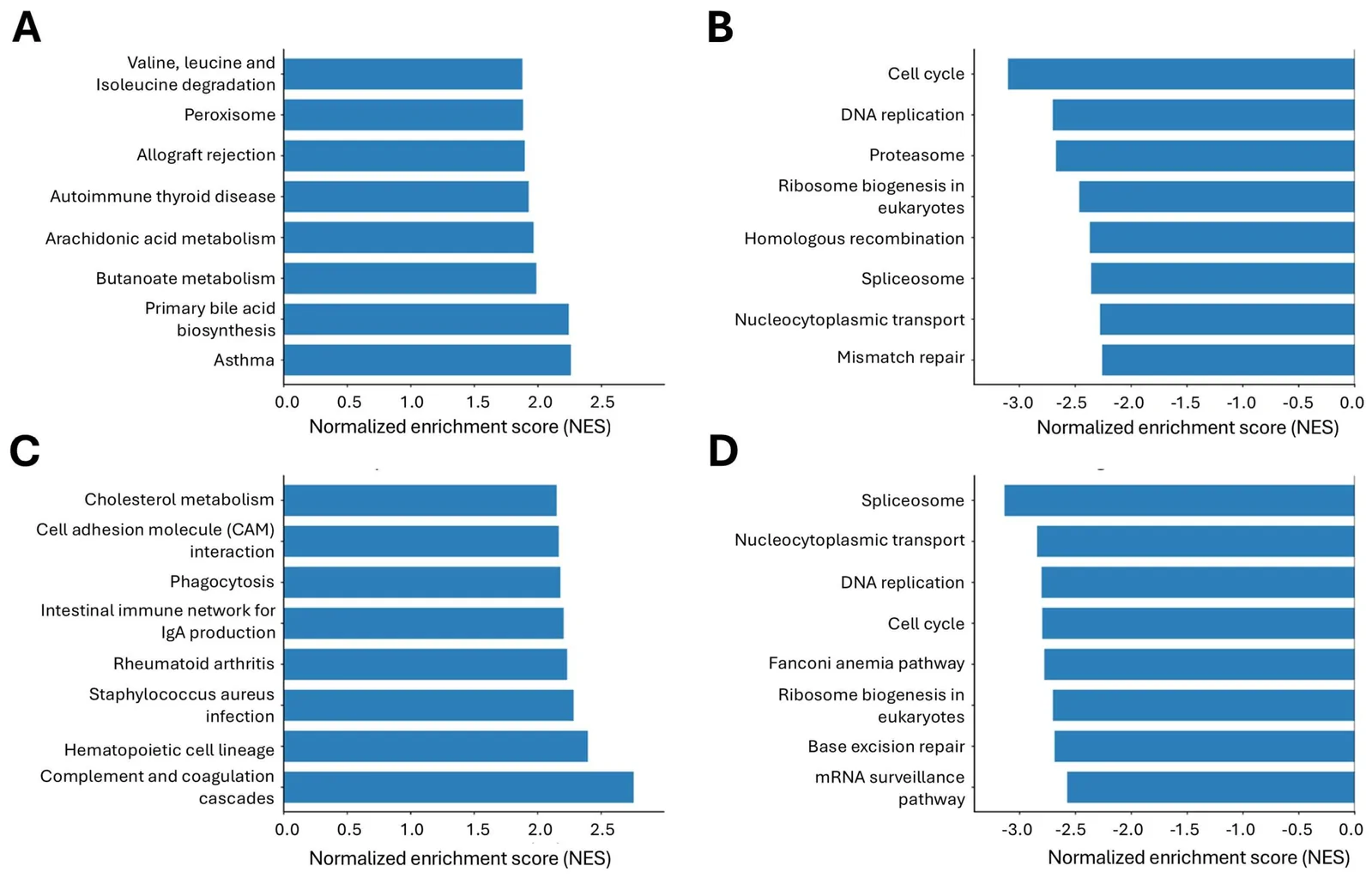
KEGG gene-set enrichment analysis of *CDKL2*-high versus *CDKL2*-low tumors in LUAD and LUSC. Within each histological subtype, tumors were divided according to the median *CDKL2* expression level, and genes were ranked using the limma-voom differential-expression statistic for the *CDKL2*-high versus *CDKL2*-low contrast. Positive normalized enrichment scores (NESs) indicate enrichment toward *CDKL2*-high tumors, whereas negative NESs indicate enrichment toward *CDKL2*-low tumors. (**A**) The eight significant positively enriched KEGG pathways with the largest absolute NES values in LUAD. (**B**) The eight significant negatively enriched pathways with the largest absolute NES values in LUAD. (**C**) The eight significant positively enriched pathways with the largest absolute NES values in LUSC. (**D**) The eight significant negatively enriched pathways with the largest absolute NES values in LUSC. Pathways with an FDR q-value < 0.05 were considered significant. Complete KEGG GSEA results are provided in Supplementary Table S3.

The KEGG GSEA identified substantial overlap in the transcriptional programs associated with *CDKL2* grouping across histologies (Figure 3; Supplementary Table S3). Because the ranking contrast was *CDKL2*-high minus *CDKL2*-low, negative NES values indicate that the pathway is enriched in *CDKL2*-low tumors.

In LUAD, the strongest negative enrichment involved the cell cycle (NES = −3.101), DNA replication (NES = −2.701), proteasome (NES = −2.672), ribosome biogenesis in eukaryotes (NES = −2.464), homologous recombination (NES = −2.369), spliceosome (NES = −2.358), nucleocytoplasmic transport (NES = −2.279), and mismatch repair (NES = −2.260); all had FDR q < 0.001. Positive enrichment toward *CDKL2*-high LUAD included asthma (NES = 2.259), primary bile acid biosynthesis (NES = 2.242), butanoate metabolism (NES = 1.988), arachidonic acid metabolism (NES = 1.965), autoimmune thyroid disease (NES = 1.927), allograft rejection (NES = 1.897), peroxisome (NES = 1.883), and valine/leucine/isoleucine degradation (NES = 1.877), all with FDR q < 0.01.

In LUSC, the strongest negative pathways were the spliceosome (NES = −3.135), nucleocytoplasmic transport (NES = −2.845), DNA replication (NES = −2.802), cell cycle (NES = −2.798), Fanconi anemia pathway (NES = −2.778), ribosome biogenesis in eukaryotes (NES = −2.702), base excision repair (NES = −2.687), and mRNA surveillance pathway (NES = −2.575); all had FDR q < 0.001. Positive enrichment toward *CDKL2*-high LUSC included complement and coagulation cascades (NES = 2.756), hematopoietic cell lineage (NES = 2.395), Staphylococcus aureus infection (NES = 2.283), rheumatoid arthritis (NES = 2.232), intestinal immune network for IgA production (NES = 2.204), phagocytosis (NES = 2.180), cell adhesion molecule interaction (NES = 2.166), and cholesterol metabolism (NES = 2.148), all with FDR q < 0.001. These analyses identify associated transcriptional programs and do not establish direct regulation by *CDKL2*.

### 3.5. CDKL2 Expression Is Associated with Molecular and Tumor Microenvironment Features

Exploratory transcript-level correlation analyses included 492 LUAD and 488 LUSC participants (Supplementary Tables S1 and S2). In LUAD, Spearman analysis showed positive correlations of *CDKL2* expression with *EGFR* (ρ = 0.361, FDR q < 0.001), *ALK* (ρ = 0.165, q < 0.001), *BRAF* (ρ = 0.403, q < 0.001), and age (ρ = 0.134, q = 0.004), whereas correlations with *KRAS* and *TP53* were not significant after FDR correction. Pearson sensitivity analyses supported the associations with *EGFR*, *BRAF*, and age, whereas the weak *ALK* association was detected only by Spearman correlation. In LUSC, only *SOX2* showed a significant inverse correlation with *CDKL2* after FDR correction (ρ = −0.146, q = 0.007), and this association was also significant by Pearson correlation (r = −0.200, q < 0.001). *TP53*, *CDKN2A*, *PIK3CA*, *NOTCH1*, and age were not significantly correlated with *CDKL2* in LUSC.

Estimated tumor purity was weakly inversely correlated with *CDKL2* expression in both LUAD (Spearman rho = −0.107, *p* = 0.020; n = 467) and LUSC (rho = −0.162, *p* < 0.001; n = 472). Adding ESTIMATE purity to the pooled Cox model left the main findings essentially unchanged: LUAD HR = 0.723 (95% CI 0.604–0.866, *p* < 0.001), LUSC HR = 1.242 (95% CI 1.009–1.528, *p* = 0.041), and *CDKL2* × histology interaction HR = 1.717 (95% CI 1.308–2.254, *p* < 0.001). The purity-adjusted model included 939 participants and 372 deaths (Supplementary Table S8A). Complementary TIMER2.0 analyses were directionally consistent and were interpreted as sensitivity analyses rather than independent replication [16].

*CDKL2* expression was also weakly positively correlated with ESTIMATE ImmuneScore in both LUAD (Spearman ρ = 0.139, *p* = 0.0025, FDR q = 0.0034; n = 471) and LUSC (ρ = 0.162, *p* < 0.001, q = 0.0015; n = 472). StromalScore was not significantly correlated with *CDKL2* in LUAD (ρ = 0.070, *p* = 0.130, q = 0.130), whereas a weak positive correlation was observed in LUSC (ρ = 0.155, *p* < 0.001, q = 0.0015).

TIMER deconvolution analysis further identified associations between *CDKL2* expression and several estimated immune-cell populations after adjustment for tumor purity. In LUAD, positive correlations were observed with B cells, CD4+ T cells, macrophages, and dendritic cells, whereas associations with CD8+ T cells and neutrophils were not statistically significant after FDR correction. In LUSC, *CDKL2* expression was positively associated with all six estimated immune-cell populations, with the largest effect observed for macrophages (partial Spearman ρ = 0.386, FDR q < 0.001). Most associations were weak in magnitude (Supplementary Table S8C). These associations reflect computationally estimated immune-cell abundance and do not establish a direct functional relationship between *CDKL2* expression and immune-cell infiltration.

### 3.6. Exploratory Single-Cell Analysis

Single-cell analysis of primary LUAD and LUSC tumors showed that *CDKL2* detection was concentrated in the epithelial compartment rather than being broadly distributed across immune or stromal populations. In LUAD, *CDKL2* was detected in 14.98% of epithelial cells, whereas detection in each non-epithelial coarse cell-type category was below 0.5%. In LUSC, epithelial cells also showed the highest *CDKL2* detection rate among the coarse cell-type categories, although the detection rate was substantially lower than in LUAD (1.57%) (Supplementary Table S10A).

Examination of epithelial annotations showed that *CDKL2* was detectable in both annotated malignant cells and other epithelial populations, indicating that *CDKL2* was not restricted to malignant cells (Supplementary Table S10B). At the donor level, among samples containing at least 50 annotated malignant cells, the median proportion of *CDKL2*-positive malignant cells was 8.48% (IQR 1.96–20.2%) in LUAD and 0.706% (IQR 0.0608–1.35%) in LUSC (Supplementary Figure S5; Supplementary Table S11). The higher malignant-cell *CDKL2* detection in LUAD remained directionally consistent when minimum thresholds of 100 and 200 malignant cells per donor were applied (Supplementary Table S12).

To reduce potential confounding by source-study differences, a within-study analysis was performed in the Wu_Zhou_2021 cohort. Among 17 LUAD and 17 LUSC donors, the median proportion of *CDKL2*-positive malignant cells was 6.59% (IQR 1.70–11.9%) and 0.706% (IQR 0.0608–1.35%), respectively (Wilcoxon *p* = 0.0013). Malignant-cell pseudobulk analysis showed concordantly higher *CDKL2* expression in LUAD than in LUSC (log2 fold change = 3.66, 95% CI 2.49–4.83; FDR-adjusted *p* = 5.41 × 10^−8^) (Supplementary Table S11).

These findings suggest histology-related differences in the cellular expression pattern of *CDKL2*, but do not establish a mechanism for the observed survival associations.

### 3.7. External Evaluation of CDKL2 in GSE30219

The GSE30219 expression cohort comprised 85 LUAD and 61 LUSC tumors. After exclusion of two LUAD cases with an OS time of 0 months, survival analyses included 83 LUAD and 61 LUSC participants, with 43 deaths in each histology. Among the survival-evaluable participants, the mean age was 61.1 ± 9.1 years in LUAD and 63.5 ± 8.7 years in LUSC. Males accounted for 65 of 83 LUAD participants (78.3%) and 56 of 61 LUSC participants (91.8%). Most participants had early pathological T and N categories, with T1 disease in 69 of 83 LUAD and 49 of 61 LUSC cases and N0 disease in 80 of 83 LUAD and 52 of 61 LUSC cases (Supplementary Table S6A,B).

**Figure 4:**
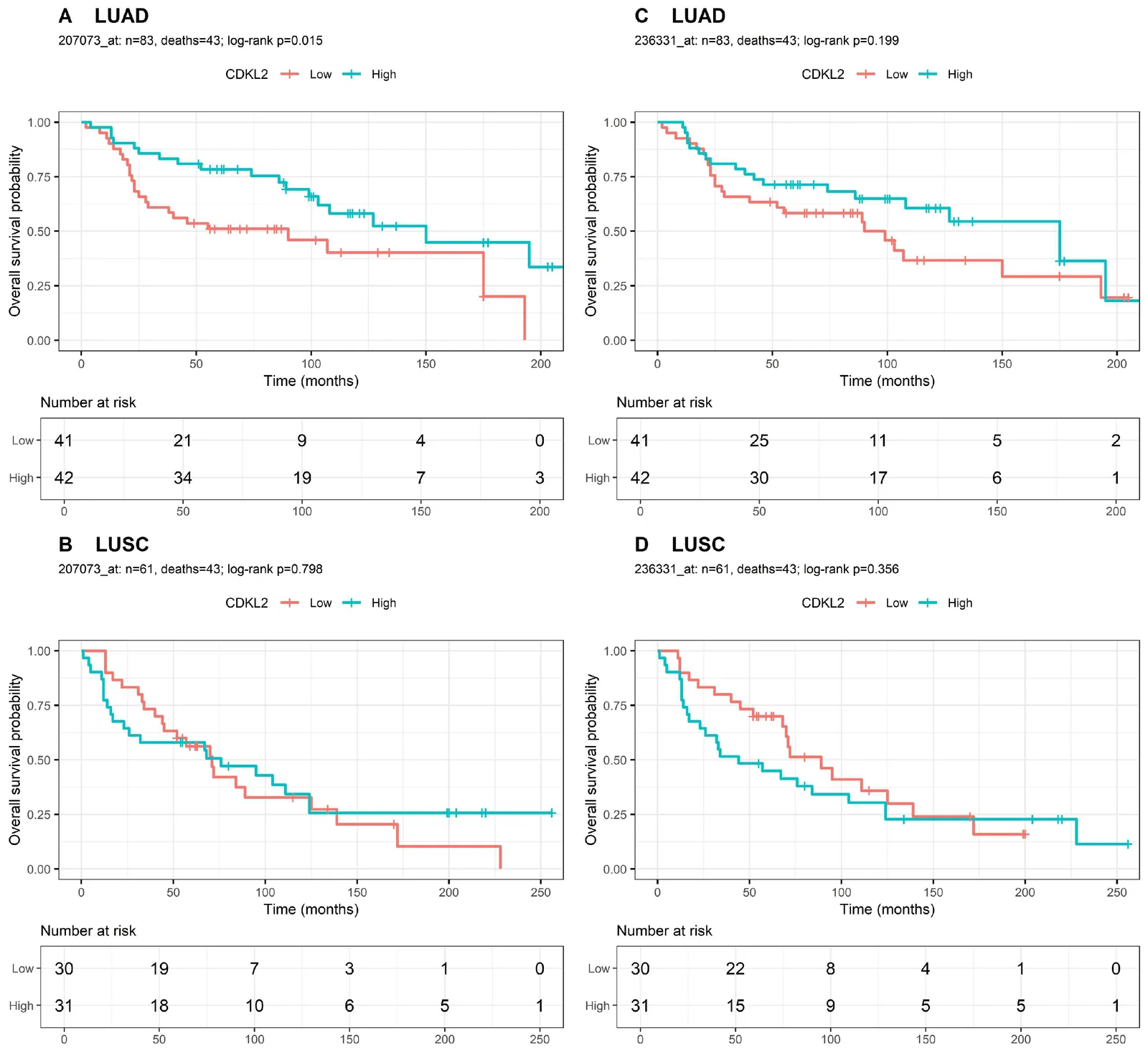
External Kaplan–Meier evaluation of overall survival according to CDKL2 expression in GSE30219 using both CDKL2-mapped probe sets. Patients were dichotomized at the histology-specific median expression value of each probe set. (**A**) 207073_at in LUAD (n = 83; 43 deaths; log-rank *p* = 0.015). (**B**) 207073_at in LUSC (n = 61; 43 deaths; log-rank *p* = 0.798). (**C**) 236331_at in LUAD (n = 83; 43 deaths; log-rank *p* = 0.199). (**D**) 236331_at in LUSC (n = 61; 43 deaths; log-rank *p* = 0.356). Numbers at risk and censoring marks are shown. Median-based adjusted Cox regression results are reported in Table 4.

Both *CDKL2*-mapped probe sets were evaluated in parallel. For 207073_at, the median-based LUAD analysis showed better OS in the *CDKL2*-high group (log-rank *p* = 0.015), and the adjusted high-versus-low Cox model was significant (HR = 0.473, 95% CI 0.250–0.897, *p* = 0.022). No corresponding association was observed in LUSC (log-rank *p* = 0.798; adjusted HR = 1.081, 95% CI 0.572–2.044, *p* = 0.810). For 236331_at, neither the LUAD analysis (log-rank *p* = 0.199; adjusted HR = 0.765, 95% CI 0.378–1.547, *p* = 0.455) nor the LUSC analysis (log-rank *p* = 0.356; adjusted HR = 1.097, 95% CI 0.546–2.205, *p* = 0.794) was statistically significant (Figure 4; Table 4).

In the probe-specific pooled continuous models, neither probe set provided significant evidence of a *CDKL2* association or histology interaction. For 207073_at, the LUAD-specific HR per 1 pooled SD was 0.727 (95% CI 0.512–1.031, *p* = 0.073), the LUSC-specific HR was 0.652 (95% CI 0.294–1.445, *p* = 0.292), and the *CDKL2* × histology interaction HR was 0.897 (95% CI 0.375–2.144, *p* = 0.806). For 236331_at, the corresponding HRs were 0.768 (95% CI 0.547–1.078, *p* = 0.126) in LUAD, 1.038 (95% CI 0.568–1.898, *p* = 0.903) in LUSC, and 1.352 (95% CI 0.685–2.672, *p* = 0.385) for the interaction (Table 4).

Sensitivity analyses using collapsed pathological T stage (T1 versus T2–4) and N stage (N0 versus N1+) did not materially alter the overall pattern. In histology-specific analyses, both probe sets retained HRs below 1 in LUAD under continuous and median-based parameterizations, but statistical significance remained confined to the median-based 207073_at model (HR = 0.480, 95% CI 0.253–0.910, *p* = 0.024); no significant association was observed in LUSC (Supplementary Table S6C). The pooled continuous interactions also remained nonsignificant after stage collapsing for both 207073_at (interaction HR = 0.841, 95% CI 0.358–1.978, *p* = 0.692) and 236331_at (interaction HR = 1.316, 95% CI 0.672–2.576, *p* = 0.424) (Supplementary Table S6D). For the collapsed-stage median-based 207073_at LUAD model, the proportional-hazards assumption was not violated (global *p* = 0.769), and the model converged without warnings or evidence of numerical instability (Supplementary Table S7A,B).

## 4. Discussion

This analysis identifies the favorable association between higher *CDKL2* expression and OS in LUAD as the most consistent clinical finding. In TCGA, continuous *CDKL2* expression was associated with a 28% lower adjusted mortality hazard per pooled SD in LUAD. This finding was supported by median-based Kaplan–Meier analysis, a graded quartile relationship, and no evidence of nonlinearity in the restricted cubic spline analysis. In GSE30219, the LUAD association showed suggestive consistency with the TCGA finding, although results varied across probe sets and modeling approaches. Taken together, these findings support a favorable prognostic association of CDKL2 in LUAD while emphasizing the need for further independent evaluation.

TCGA also provided strong statistical evidence that the *CDKL2*-OS association differed by histology. The continuous *CDKL2* × histology interaction was significant and remained so in separate sensitivity analyses using stratification for variables showing nonproportional hazards and adjustment for estimated tumor purity. However, formal heterogeneity was not supported in GSE30219 for either probe set. The probe-specific estimates remained nonsignificant after collapsing sparse T- and N-stage categories, suggesting that sparse stage categories alone were unlikely to explain the nonsignificant external interaction. The external LUSC cohort was small, and its stage distribution was concentrated in T1/N0 disease. In addition, TCGA and GSE30219 differ in transcriptomic platform, patient selection, treatment history, follow-up, clinical annotation, and available covariates for multivariable adjustment. These factors may reduce statistical power and limit the reproducibility of effect estimates across cohorts. The adverse LUSC association should therefore be regarded as a TCGA finding that remains preliminary rather than an established opposite prognostic effect.

The pathway analysis offers biological context but does not resolve the survival discrepancy by itself. In both LUAD and LUSC, *CDKL2*-low tumors were strongly enriched for cell-cycle, DNA-replication, and genome-maintenance programs. These pathway-level findings link lower *CDKL2* expression with greater enrichment of proliferation-related transcriptional programs. Nevertheless, similar pathway-level signatures can have different prognostic consequences in different histologic contexts because LUAD and LUSC have distinct molecular backgrounds and tumor-microenvironmental characteristics [3,4]. Histology-specific prognostic expression effects have also been reported more broadly in NSCLC [22]. Thus, these findings suggest that the prognostic effect of *CDKL2* may depend on histologic context, while the biological basis for this difference remains unresolved.

Positive enrichment of immune- and microenvironment-related pathways, particularly complement/coagulation and hematopoietic programs in LUSC, provides additional context for the bulk-tumor findings. Complement and coagulation systems can interact with immune, vascular, and stromal components of the tumor microenvironment [23,24]. In the present data, *CDKL2* expression showed weak inverse correlations with estimated tumor purity and weak positive associations with ESTIMATE ImmuneScore in both histologies. TIMER deconvolution similarly showed weak associations between *CDKL2* expression and several estimated immune-cell populations. These findings suggest that bulk-tumor *CDKL2* expression covaries with features of the tumor microenvironment, but they do not establish that immune cells are a major source of *CDKL2* expression or that *CDKL2* directly regulates immune-cell recruitment.

Importantly, adding ESTIMATE purity to the primary TCGA Cox model produced nearly unchanged *CDKL2* estimates and an essentially identical interaction effect, indicating that adjustment for overall tumor purity did not materially alter the TCGA survival association. Complementary single-cell analysis further showed that *CDKL2* detection was concentrated predominantly in epithelial populations rather than being broadly distributed across immune or stromal compartments. However, *CDKL2* was detected in both malignant and non-malignant epithelial populations and should therefore not be interpreted as a tumor-cell-specific marker. Taken together, the bulk and single-cell analyses provide complementary information on the cellular context of *CDKL2* expression, but they do not establish a direct relationship between epithelial *CDKL2* expression and the tumor microenvironment. The higher malignant-cell *CDKL2* detection observed in LUAD than in LUSC, including within the Wu_Zhou_2021 cohort, further suggests histology-related differences in cellular expression context; whether these differences contribute to the distinct prognostic associations remains to be determined.

The tumor–normal analysis also illustrates the importance of reference selection. A TCGA-only GDC analysis demonstrated lower *CDKL2* expression in tumor tissue than in normal lung in both LUAD and LUSC, including paired-sample sensitivity analyses. By contrast, the earlier GEPIA2 TCGA/GTEx framework did not show a significant LUAD difference [11]. Because TCGA and GTEx tissues differ in acquisition, processing, and donor composition, the discrepancy is plausibly attributable to differences in the normal reference framework rather than to a simple biological contradiction. For the manuscript, the TCGA-only comparison is therefore emphasized, while GEPIA2 is retained as complementary pan-cancer context.

The GSE30219 analysis also highlights important limitations. Across both CDKL2-mapped probe sets, LUAD effect estimates were directionally favorable under continuous and median-based parameterizations after clinical adjustment, but statistical significance was confined to the median-based 207073_at model. This association remained materially unchanged after collapsing sparse T- and N-stage categories, suggesting that the result was not driven solely by sparse stage categorization. Median dichotomization can accentuate contrasts while sacrificing information and generalizability. The cohort-specific median cutoff should therefore not be interpreted as a clinically transferable threshold. The external LUAD evidence should thus be considered limited and suggestive, with sensitivity to both probe selection and expression parameterization.

Several limitations remain. First, this is a retrospective analysis of public datasets, and no causal inference can be made. Selection bias related to tissue availability, molecular profiling, clinical annotation, and survival-data completeness may also limit the generalizability of the study populations. Second, GSE30219 is substantially smaller than TCGA and differs in assay platform and clinical composition, with sparse advanced-stage categories and limited covariate availability for adjustment. Third, the principal prognostic measurements are based on bulk-tissue mRNA expression. Although exploratory single-cell analysis provided information on the cellular distribution of *CDKL2*, these findings were derived from an integrated public atlas and do not establish cell-type-specific function. Protein-level relevance also remains unresolved. We additionally examined the harmonized quantitative proteome matrices of the CPTAC LUAD and LUSC discovery studies [25,26]; however, CDKL2 was not represented in either dataset, precluding reliable protein-level validation. Protein-level assessment using alternative proteomic or experimental approaches therefore remains an important direction for future investigation. Fourth, the exploratory transcript correlations should not be interpreted as genomic alteration analyses. Fifth, GSEA identifies coordinated transcriptional associations and cannot establish that *CDKL2* directly regulates cell-cycle, DNA-repair, or immune pathways [15]. Finally, we did not assess incremental clinical prediction performance such as change in C-index, time-dependent AUC, calibration, or decision-curve benefit. Accordingly, *CDKL2* should be described as a candidate prognostically associated marker rather than a clinically validated prognostic biomarker.

Despite these limitations, the analyses support a favorable association between higher *CDKL2* expression and OS in LUAD. The primary TCGA model uses continuous expression and a formal histology interaction, and the finding is supported by proportional-hazards diagnostics, spline- and quartile-based sensitivity analyses, and tumor-purity adjustment. GSE30219 provided additional suggestive evidence for the LUAD association, although the strength of this evidence varied across probe sets and modeling approaches. These results justify further investigation of *CDKL2* in LUAD while emphasizing that the LUSC association requires evaluation in larger independent cohorts.

## 5. Conclusions

Higher *CDKL2* expression was associated with favorable OS in TCGA-LUAD, with consistent findings across continuous modeling, quartile sensitivity analysis, and tumor-purity adjustment. In GSE30219, the LUAD association was directionally consistent with the TCGA finding, although statistical support varied across probe sets and modeling approaches.

TCGA demonstrated a significant *CDKL2* × histology interaction, indicating statistical heterogeneity between LUAD and LUSC. However, neither the adverse LUSC association nor the formal interaction was supported in GSE30219, including after sensitivity analysis with collapsed T- and N-stage categories. The LUSC finding should therefore remain preliminary.

*CDKL2* grouping was associated with proliferation-, DNA-replication-, DNA-maintenance-, metabolic-, and immune-related transcriptional programs; however, these findings are associative and hypothesis-generating. Additional independent cohorts, cell-type-resolved analyses, protein-level validation, and functional studies are needed before the mechanistic or clinical relevance of *CDKL2* in NSCLC can be established.

## Figures and Tables

**Table 1:** Clinical characteristics by *CDKL2* expression in TCGA-LUAD.

		*CDKL2*		
	Low, n (%)	High, n (%)	Missing Low/High	*p*-Value
Age				
≤65	126 (51.2%)	111 (45.1%)	0/0	0.176
>65	120 (48.8%)	135 (54.9%)		
Sex				
Female	123 (50.0%)	144 (58.5%)	0/0	0.057
Male	123 (50.0%)	102 (41.5%)		
Smoking Status				
Ever	215 (90.7%)	193 (80.1%)	9/5	0.001
Never	22 (9.3%)	48 (19.9%)		
Pathologic Stage				
Stage I	124 (51.0%)	138 (57.3%)	3/5	0.323
Stage II	61 (25.1%)	57 (23.7%)		
Stage III/IV	58 (23.9%)	46 (19.1%)		

Note: Percentages are calculated among non-missing observations within each *CDKL2* group. All comparisons used Pearson chi-square tests. Clinicopathological analyses were exploratory.

**Table 2:** Clinical characteristics by *CDKL2* expression in TCGA-LUSC.

		*CDKL2*		
	Low, n (%)	High, n (%)	Missing Low/High	*p*-Value
Age				
≤65	101 (41.4%)	88 (36.1%)	0/0	0.227
>65	143 (58.6%)	156 (63.9%)		
Sex				
Female	54 (22.1%)	73 (29.9%)	0/0	0.050
Male	190 (77.9%)	171 (70.1%)		
Smoking Status				
Ever	230 (97.0%)	228 (95.4%)	7/5	0.346
Never	7 (3.0%)	11 (4.6%)		
Pathologic Stage				
Stage I	102 (42.0%)	137 (56.8%)	1/3	0.002
Stage II	95 (39.1%)	61 (25.3%)		
Stage III/IV	46 (18.9%)	43 (17.8%)		

Note: Percentages are calculated among non-missing observations within each *CDKL2* group. All comparisons used Pearson chi-square tests. Clinicopathological analyses were exploratory.

**Table 3:** Multivariable Cox proportional-hazards analysis of overall survival in the pooled TCGA LUAD/LUSC cohort (n = 943; 375 deaths).

Variable	HR	95% CI	*p*-Value
*CDKL2* per 1 pooled SD in LUAD	0.721	0.603–0.862	<0.001
*CDKL2* per 1 pooled SD in LUSC	1.237	1.011–1.515	0.039
*CDKL2* × LUSC interaction	1.717	1.313–2.246	<0.001
LUSC vs. LUAD at *CDKL2* z = 0	0.992	0.767–1.283	0.952
Age, per year	1.019	1.007–1.031	0.002
Male vs. female	1.152	0.923–1.438	0.212
Stage II vs. stage I	1.530	1.194–1.962	<0.001
Stage III/IV vs. stage I	2.326	1.815–2.981	<0.001
Ever vs. never smoker	0.778	0.529–1.145	0.202

Note: *CDKL2* was modeled continuously per one pooled SD. LUAD, female sex, stage I, and never smoking were reference categories. The interaction HR is the multiplicative ratio of the LUSC *CDKL2* hazard ratio to the LUAD *CDKL2* hazard ratio.

**Table 4:** External evaluation of overall survival in GSE30219.

Probe Set	Model	Effect/Histology	N	Deaths	Log-Rank *p*-Value	Adjusted HR	95% CI	Cox *p*-Value
207073_at	Median high vs. low	LUAD	83	43	0.015	0.473	0.250–0.897	0.022
	Median high vs. low	LUSC	61	43	0.798	1.081	0.572–2.044	0.810
	Continuous pooled interaction model	LUAD effect	144	86	N/A	0.727	0.512–1.031	0.073
	Continuous pooled interaction model	LUSC effect	144	86	N/A	0.652	0.294–1.445	0.292
	Continuous pooled interaction model	*CDKL2* × histology	144	86	N/A	0.897	0.375–2.144	0.806
236331_at	Median high vs. low	LUAD	83	43	0.199	0.765	0.378–1.547	0.455
	Median high vs. low	LUSC	61	43	0.356	1.097	0.546–2.205	0.794
	Continuous pooled interaction model	LUAD effect	144	86	N/A	0.768	0.547–1.078	0.126
	Continuous pooled interaction model	LUSC effect	144	86	N/A	1.038	0.568–1.898	0.903
	Continuous pooled interaction model	*CDKL2* × histology	144	86	N/A	1.352	0.685–2.672	0.385

Note: Both currently annotated *CDKL2*-mapped probe sets, 207073_at and 236331_at, were analyzed in parallel. Median-based analyses used the histology-specific median expression cutoff for each probe set and were adjusted for age, sex, pathological T stage, and pathological N stage. For each probe set, continuous expression was standardized across the pooled LUAD/LUSC survival cohort, and the pooled model included standardized *CDKL2* expression, histologic subtype, the *CDKL2* × histology interaction, age, sex, pathological T stage, and pathological N stage. LUAD was the reference histology. Histology-specific continuous effects were derived from the corresponding probe-specific pooled model. N/A indicates that a log-rank *p*-value is not applicable to the continuous pooled interaction model.

## Data Availability

The datasets analyzed in this study are publicly available. TCGA-LUAD and TCGA-LUSC *CDKL2* expression data used for the survival analyses were obtained from OncoLnc. Overall survival (OS), progression-free interval (PFI), age, sex, and pathologic stage annotations were obtained from the TCGA Pan-Cancer Clinical Data Resource, whereas smoking history was obtained from the PanCanAtlas clinical follow-up dataset. TCGA RNA-seq data used for tumor-versus-normal expression and exploratory transcript-correlation analyses were obtained from the Genomic Data Commons using TCGAbiolinks. ESTIMATE tumor-purity estimates used in the purity-adjusted survival analyses were obtained from the TCGA tumor-purity dataset reported by Aran et al. Precomputed ESTIMATE-derived ImmuneScore and StromalScore values for TCGA-LUAD and TCGA-LUSC were obtained from the ESTIMATE disease-centric resource (RNA-seqV2; accessed 16 August 2026) and used for tumor-microenvironment analyses. The independent GSE30219 dataset is publicly available from the NCBI Gene Expression Omnibus under accession number GSE30219. GEPIA2 and TIMER2.0 were used as complementary public web-based resources. The LuCA core single-cell atlas used for exploratory cellular-source analyses was obtained from CZ CELLxGENE. The Wu_Zhou_2021 cohort was analyzed as a source-study subset within the LuCA atlas for a within-study analysis. The complete analysis scripts and key processed analysis outputs are provided in Supplementary File S1, together with a README file describing the input files and execution order.

## Supplementary Materials

The following supporting information can be downloaded at: https://www.mdpi.com/article/10.3390/medicina62091638/s1, Figure S1: Pan-cancer *CDKL2* expression in tumor and reference normal tissues using GEPIA2; Figure S2: Pan-cancer Cox proportional-hazards analysis of *CDKL2* expression using TIMER2.0; Figure S3: Pan-cancer tumor-purity-adjusted mRNA expression correlations between *CDKL2* and selected lung cancer-related genes using TIMER2.0; Figure S4: Schoenfeld residual diagnostics for the primary TCGA Cox proportional-hazards model; Figure S5: Donor-level detection of *CDKL2* in annotated malignant cells from primary LUAD and LUSC tumors in the LuCA core atlas; Figure S6: Kaplan–Meier analysis of progression-free interval according to *CDKL2* expression in TCGA LUAD and LUSC; Table S1: Exploratory Spearman and Pearson correlations of *CDKL2* expression with selected variables in LUAD; Table S2: Exploratory Spearman and Pearson correlations of *CDKL2* expression with selected variables in LUSC; Table S3: Complete KEGG GSEA results for *CDKL2*-associated transcriptional profiles in LUAD and LUSC; Table S4: Patient selection for the primary TCGA Cox analysis; Table S5A–D: Proportional-hazards diagnostics for the primary TCGA Cox model, sex- and pathologic-stage-stratified Cox sensitivity analysis, restricted cubic spline tests for nonlinearity, and quartile-based TCGA sensitivity analyses; Table S6A–D: GSE30219 cohort characteristics, collapsed pathological T/N stage distribution, two-probe adjusted survival analyses, and probe-specific pooled continuous *CDKL2* × histology interaction analyses; Table S7A,B: Collapsed-stage median-based LUAD Cox analysis using probe set 207073_at and corresponding proportional-hazards/model-stability diagnostics; Table S8A–C: Tumor-purity-adjusted pooled TCGA Cox model, ESTIMATE ImmuneScore/StromalScore associations, and TIMER immune-cell deconvolution analyses; Table S9: TCGA-only tumor-versus-normal expression sensitivity analysis; Table S10A,B: Cellular distribution of *CDKL2* detection across coarse cell-type and epithelial annotations in primary LUAD and LUSC tumors in the LuCA core atlas; Table S11: Donor-level and within-study analyses of malignant-cell *CDKL2* detection and expression; Table S12: Sensitivity analysis of donor-level malignant-cell *CDKL2* detection using different minimum malignant-cell thresholds; Table S13A,B: Proportional-hazards diagnostics and stage-stratified sensitivity analysis for progression-free interval; Supplementary File S1: Reproducibility package containing the complete analysis scripts, a README describing the required input data, software requirements, and recommended execution order, the renv.lock file recording the final R package environment, and key processed analysis outputs generated by the analysis scripts.
