# Supplementary material for: Prognostic Significance of CDKL2 in Non-Small Cell Lung Cancer: A Favorable Association in Lung Adenocarcinoma
Source: Medicina (Kaunas). 2026 Aug 27;62(9):1638. doi: 10.3390/medicina62091638 (PMC13609535; doi:10.3390/medicina62091638)
Supplement: Supplementary file 1 [file medicina-62-01638-s001.zip › medicina-4481099-supplementary.pdf]

Supplementary Materials

Supplementary Table S1. Exploratory Spearman and Pearson correlations of *CDKL2* expression with selected variables in LUAD.

Variable	N	Spearman ρ	p-value	FDR q-value	Pearson r	p-value	FDR q-value
EGFR	492	0.361	< 0.001	< 0.001	0.318	< 0.001	< 0.001
KRAS	492	0.080	0.076	0.091	0.040	0.379	0.391
TP53	492	0.050	0.265	0.265	0.070	0.120	0.179
ALK	492	0.165	< 0.001	< 0.001	0.039	0.391	0.391
BRAF	492	0.403	< 0.001	< 0.001	0.429	< 0.001	< 0.001
Age	492	0.134	0.003	0.004	0.147	0.001	0.002

Note: Correlations were calculated using participant-level log2(TPM + 1) expression values from GDC primary-tumor RNA-seq data. When multiple primary-tumor RNA-seq aliquots were available for the same participant, transformed expression values were averaged at the participant level. Spearman rank correlation was used as the primary analysis, with Pearson correlation performed as a sensitivity analysis. Benjamini-Hochberg correction was applied separately across the six comparisons within each histologic subtype and correlation method. Gene variables represent transcript-level mRNA co-expression, whereas age was evaluated as an exploratory clinical correlate. These analyses were not adjusted for tumor purity.

Supplementary Table S2. Exploratory Spearman and Pearson correlations of *CDKL2* expression with selected variables in LUSC.

Variable	N	Spearman ρ	p-value	FDR q-value	Pearson r	p-value	FDR q-value
TP53	488	0.019	0.672	0.775	0.007	0.885	0.928
CDKN2A	488	-0.051	0.259	0.775	-0.054	0.233	0.700
SOX2	488	-0.146	0.001	0.007	-0.200	< 0.001	< 0.001
PIK3CA	488	0.032	0.475	0.775	-0.010	0.821	0.928
NOTCH1	488	0.013	0.775	0.775	0.019	0.679	0.928
Age	488	0.028	0.532	0.775	0.004	0.928	0.928

Note: Correlations were calculated using participant-level log2(TPM + 1) expression values from GDC primary-tumor RNA-seq data. When multiple primary-tumor RNA-seq aliquots were available for the same participant, transformed expression values were averaged at the participant level. Spearman rank correlation was used as the primary analysis, with Pearson correlation performed as a sensitivity analysis. Benjamini-Hochberg correction was applied separately across the six comparisons within each histologic subtype and correlation method. Gene variables represent transcript-level mRNA co-expression, whereas age was evaluated as an exploratory clinical correlate. These analyses were not adjusted for tumor purity.

Supplementary Table S3. Complete KEGG GSEA results for *CDKL2*-associated transcriptional profiles in LUAD and LUSC.

The table contains the complete results for all 354 tested KEGG pathways in each histological subtype, including pathway name, normalized enrichment score (NES), nominal p-value, adjusted p-

value, FDR q-value, gene-set size, and leading-edge genes. Supplementary Table S3 is provided as a separate Excel file.

Supplementary Table S4. Patient selection for the primary TCGA Cox analysis.

Selection step	LUAD	LUSC	Total
Patients with available *CDKL2* expression	492	488	980
Excluded: missing smoking only	13	12	25
Excluded: missing pathologic stage only	7	4	11
Excluded: both smoking and stage missing	1	0	1
Total excluded	21	16	37
Primary complete-case cohort	471	472	943
Deaths in primary cohort	168	207	375

Supplementary Table S5A. Proportional-hazards diagnostics for the primary TCGA Cox model.

Term	Chi-square	df	p-value
CDKL2 expression	3.567	1	0.059
Histology	2.380	1	0.123
Age	0.275	1	0.600
Sex	4.000	1	0.045
Pathologic stage	9.201	2	0.010
Smoking status	2.192	1	0.139
CDKL2 × histology interaction	0.002	1	0.962
GLOBAL	19.634	8	0.012

Note: Schoenfeld-residual tests are diagnostic tests and therefore do not produce hazard ratios or confidence intervals.

Supplementary Table S5B. Sex- and pathologic-stage-stratified sensitivity Cox model.

Variable	HR	95% CI	p-value
CDKL2 per 1 pooled SD in LUAD	0.703	0.588–0.841	< 0.001
LUSC vs LUAD at *CDKL2* z = 0	0.992	0.766–1.285	0.952
Age, per year	1.019	1.007–1.031	0.002
Ever vs never smoker	0.780	0.528–1.152	0.211
CDKL2 × histology interaction	1.764	1.350–2.305	< 0.001

Note: Sex and pathologic stage were used as stratification variables and therefore have no estimated HRs in this model. The global Schoenfeld test was nonsignificant (p = 0.378), and the interaction-specific proportional-hazards test was also nonsignificant (p = 0.663).

Supplementary Table S5C. Restricted cubic spline tests for nonlinearity.

Histology	p for nonlinearity	Interpretation
LUAD	0.760	No evidence of nonlinearity
LUSC	0.991	No evidence of nonlinearity

Supplementary Table S5D. Quartile-based TCGA sensitivity analyses.

Histology	Quartile/Trend	Adjusted HR	95% CI	p-value
LUAD	Q1	1.000	Reference	N/A
LUAD	Q2	0.877	0.584–1.316	0.526
LUAD	Q3	0.557	0.366–0.847	0.006
LUAD	Q4	0.477	0.301–0.756	0.002
LUAD	Per-quartile trend	0.765	0.664–0.881	< 0.001
LUSC	Q1	1.000	Reference	N/A
LUSC	Q2	1.076	0.718–1.614	0.722
LUSC	Q3	1.300	0.873–1.936	0.197
LUSC	Q4	1.429	0.950–2.150	0.086
LUSC	Per-quartile trend	1.135	0.998–1.292	0.054

Supplementary Table S6A. GSE30219 cohort characteristics.

Histology	Expression N	Survival N	Deaths	Censored	Age, mean ± SD (years)	Male, n (%)	Female, n (%)	Median follow-up (months)
LUAD	85	83	43	40	61.1 ± 9.1	65 (78.3%)	18 (21.7%)	116
LUSC	61	61	43	18	63.5 ± 8.7	56 (91.8%)	5 (8.2%)	199

Note: Expression N denotes all expression-available tumors. Survival N excludes two LUAD cases with an OS time of 0 months. Age and sex summaries were calculated among survival-evaluable participants. Median follow-up was estimated using the reverse Kaplan-Meier method.

Supplementary Table S6B. Collapsed pathological T/N stage distribution in the GSE30219 survival cohort.

Histology	Category	N
LUAD	T1	69
LUAD	T2–4	14
LUAD	N0	80
LUAD	N1+	3
LUSC	T1	49
LUSC	T2–4	12
LUSC	N0	52
LUSC	N1+	9

Note: Collapsed stage coding was T1 versus T2–4 and N0 versus N1+. These categories were used in sensitivity analyses because advanced T- and N-stage categories were sparse.

Supplementary Table S6C. Adjusted histology-specific continuous and median-based Cox analyses of CDKL2 and overall survival in GSE30219.

Probe set	Histology	Parameterization	N	Deaths	Adjusted HR	95% CI	p-value
207073_at	LUAD	Continuous, per SD	83	43	0.700	0.469–1.044	0.080
207073_at	LUAD	Median high vs low	83	43	0.480	0.253–0.910	0.024
236331_at	LUAD	Continuous, per SD	83	43	0.763	0.529–1.101	0.148
236331_at	LUAD	Median high vs low	83	43	0.773	0.383–1.562	0.474
207073_at	LUSC	Continuous, per SD	61	43	0.778	0.535–1.130	0.188
207073_at	LUSC	Median high vs low	61	43	1.002	0.538–1.869	0.994
236331_at	LUSC	Continuous, per SD	61	43	0.971	0.705–1.339	0.858
236331_at	LUSC	Median high vs low	61	43	1.095	0.550–2.181	0.797

Note: Both CDKL2-mapped probe sets (207073_at and 236331_at) were analyzed in parallel. Within each histology, models were adjusted for age, sex, collapsed pathological T stage (T1 versus T2–4), and collapsed pathological N stage (N0 versus N1+). Continuous expression was standardized within histology, whereas median-based analyses used the histology-specific median for each probe set.

Supplementary Table S6D. Probe-specific pooled continuous CDKL2 × histology interaction models with collapsed T/N stage coding.

Probe set	Effect	N	Deaths	HR	95% CI	p-value
207073_at	LUAD effect	144	86	0.744	0.528–1.050	0.092
207073_at	LUSC effect	144	86	0.626	0.287–1.364	0.239
207073_at	CDKL2 × histology	144	86	0.841	0.358–1.978	0.692
236331_at	LUAD effect	144	86	0.797	0.573–1.109	0.178
236331_at	LUSC effect	144	86	1.048	0.579–1.898	0.876
236331_at	CDKL2 × histology	144	86	1.316	0.672–2.576	0.424

Note: Each probe set was analyzed separately in the pooled LUAD/LUSC survival cohort (n = 144; 86 deaths). Expression was standardized across the pooled cohort separately for each probe set. Models included standardized CDKL2 expression, histologic subtype, the CDKL2 × histology interaction, age, sex, collapsed pathological T stage (T1 versus T2–4), and collapsed pathological N stage (N0 versus N1+). LUAD was the reference histology. Full model coefficients and proportional-hazards diagnostics are retained in Supplementary File S1.

Supplementary Table S7A. Collapsed-stage median-based multivariable Cox analysis of overall survival in GSE30219 LUAD using probe set 207073_at.

Variable	Adjusted HR	95% CI	p-value
CDKL2 high vs low	0.480	0.253–0.910	0.024
Age, per year	1.028	0.992–1.065	0.127
Male vs female	0.946	0.434–2.063	0.890

Variable	Adjusted HR	95% CI	p-value
T2–4 vs T1	1.687	0.795–3.581	0.173
N1+ vs N0	0.603	0.127–2.871	0.525

Note: The model included 83 LUAD participants and 43 deaths. *CDKL2* expression was dichotomized at the LUAD-specific median. T stage was collapsed as T1 versus T2–4 and N stage as N0 versus N1+. Female sex, T1, N0, and *CDKL2*-low were the reference categories.

Supplementary Table S7B. Proportional-hazards and numerical-stability diagnostics for the collapsed-stage median-based GSE30219 LUAD Cox model using probe set 207073_at.

PH term	Schoenfeld p-value	Stability diagnostic	Result
CDKL2 high vs low	0.583	Iterations	4
Age	0.287	All coefficients finite	Yes
Sex	0.516	All standard errors finite	Yes
Collapsed T stage	0.696	Maximum absolute β	0.734
Collapsed N stage	0.536	HR range	0.480–1.687
Global	0.769	Cox model warnings	None

Note: No term showed evidence of violation of the proportional-hazards assumption, and the global test was nonsignificant (p = 0.769). The model converged without warnings; all coefficients and standard errors were finite, with no evidence of extreme coefficient estimates. Because only three LUAD participants were classified as N1+, the N-stage estimate remains imprecise despite numerical stability.

Supplementary Table S8A. Tumor-purity-adjusted pooled TCGA Cox model.

Variable	HR	95% CI	p-value
CDKL2 per 1 pooled SD in LUAD	0.723	0.604–0.866	< 0.001
CDKL2 per 1 pooled SD in LUSC	1.242	1.009–1.528	0.041
CDKL2 × LUSC interaction	1.717	1.308–2.254	< 0.001
LUSC vs LUAD at *CDKL2* z = 0	1.001	0.773–1.296	0.995
Age, per year	1.019	1.007–1.031	0.002
Male vs female	1.144	0.914–1.432	0.240
Stage II vs stage I	1.504	1.171–1.931	0.001
Stage III/IV vs stage I	2.305	1.797–2.956	< 0.001
Ever vs never smoker	0.788	0.533–1.166	0.234
ESTIMATE tumor purity	1.095	0.526–2.282	0.808

Note: The model included 939 participants and 372 deaths and was adjusted for continuous standardized *CDKL2* expression, histologic subtype, the *CDKL2* × histology interaction, age, sex, pathologic stage, smoking status, and ESTIMATE-derived tumor purity. The LUAD-specific *CDKL2* effect corresponds to the *CDKL2* main-effect coefficient, whereas the LUSC-specific effect was derived from the sum of the *CDKL2* main-effect and interaction coefficients. Stage I, female sex, never-smoking status, and LUAD were the reference categories.

Supplementary Table S8B. Associations of *CDKL2* expression with ESTIMATE ImmuneScore and StromalScore.

Histology	Metric	n	Spearman rho	p-value	FDR q-value
LUAD	ImmuneScore	471	0.139	0.0025	0.0034
LUAD	StromalScore	471	0.070	0.130	0.130
LUSC	ImmuneScore	472	0.162	< 0.001	0.0015
LUSC	StromalScore	472	0.155	< 0.001	0.0015

Note: Spearman correlations were calculated separately within LUAD and LUSC. Benjamini-Hochberg correction was applied across the four histology-score comparisons. ESTIMATE scores represent computationally inferred features of bulk-tumor microenvironmental composition.

Supplementary Table S8C. Tumor-purity-adjusted associations between *CDKL2* expression and TIMER-estimated immune-cell populations

Histology	Immune-cell population	Partial Spearman rho	p-value	FDR q-value
LUAD	B cell	0.224	6.45×10^{-7}	2.23×10^{-6}
LUAD	CD8+ T cell	0.080	0.0779	0.0850
LUAD	CD4+ T cell	0.170	1.75×10^{-4}	3.50×10^{-4}
LUAD	Macrophage	0.123	0.00676	0.00901
LUAD	Neutrophil	0.016	0.730	0.730
LUAD	Dendritic cell	0.246	3.67×10^{-8}	2.20×10^{-7}
LUSC	B cell	0.112	0.0149	0.0178
LUSC	CD8+ T cell	0.225	7.42×10^{-7}	2.23×10^{-6}
LUSC	CD4+ T cell	0.137	0.00270	0.00405
LUSC	Macrophage	0.386	1.96×10^{-18}	2.35×10^{-17}
LUSC	Neutrophil	0.138	0.00255	0.00405
LUSC	Dendritic cell	0.182	6.86×10^{-5}	1.65×10^{-4}

Note: Immune-cell abundance was estimated using the TIMER algorithm. Reported coefficients are tumor-purity-adjusted partial Spearman correlations. Benjamini-Hochberg correction was applied across the 12 histology-cell-type comparisons. These computational estimates represent associations with

inferred immune-cell abundance and do not identify the cellular source of *CDKL2* expression.

Supplementary Table S9. TCGA-only tumor-normal expression sensitivity analysis.

Histology	Tumor N	Normal N	Paired N	Tumor median	Normal median	Paired Wilcoxon p	BH-FDR
LUAD	517	59	58	2.957	3.849	7.83×10^{-7}	7.83×10^{-7}
LUSC	501	51	51	1.161	3.519	6.34×10^{-10}	1.27×10^{-9}

Note: Expression was analyzed as log2(TPM + 1) from the tpm_unstrand assay. When multiple RNA-seq aliquots of the same sample type were available for a participant, transformed expression values were averaged at the participant level. Formal tumor-normal comparisons were performed using paired Wilcoxon signed-rank tests in participants with matched specimens. Benjamini-Hochberg correction was applied across the two histology-specific paired comparisons.

Supplementary Table S10A. Cellular distribution of *CDKL2* detection across coarse cell-type annotations in primary LUAD and LUSC tumors in the LuCA core atlas.

Histology	Cell type	Total cells	*CDKL2*-positive cells	*CDKL2*-positive cells (%)
LUAD	Epithelial cell	43,369	6,497	14.981
LUAD	cDC	9,189	40	0.435
LUAD	T cell	66,191	284	0.429
LUAD	Plasma cell	5,492	23	0.419
LUAD	Endothelial cell	6,176	21	0.340
LUAD	Macrophage/Monocyte	34,042	105	0.308
LUAD	Stromal	4,750	13	0.274
LUAD	Neutrophils	1,490	3	0.201
LUAD	Mast cell	4,066	4	0.098
LUAD	B cell	12,232	12	0.098
LUAD	NK cell	4,456	4	0.090
LUAD	pDC	609	0	0.000
LUSC	Epithelial cell	33,186	520	1.567
LUSC	T cell	8,411	65	0.773
LUSC	cDC	1,723	3	0.174
LUSC	Plasma cell	2,354	4	0.170
LUSC	B cell	1,203	2	0.166
LUSC	NK cell	1,355	2	0.148
LUSC	Mast cell	695	1	0.144
LUSC	Neutrophils	3,940	5	0.127
LUSC	Macrophage/Monocyte	10,736	13	0.121
LUSC	Stromal	3,320	3	0.090

Histology	Cell type	Total cells	*CDKL2*-positive cells	*CDKL2*-positive cells (%)
LUSC	Endothelial cell	7,589	2	0.026
LUSC	pDC	331	0	0.000

Note: Analyses were restricted to cells derived from primary LUAD or LUSC tumors. *CDKL2*-positive cells were defined as cells with detectable *CDKL2* counts (> 0). Existing LuCA cell-type annotations were retained without re-clustering or re-annotation.

Supplementary Table S10B. *CDKL2* detection across epithelial annotations in primary LUAD and LUSC tumors in the LuCA core atlas.

Histology	Cell type	Total cells	*CDKL2*-positive cells	*CDKL2*-positive cells (%)
LUAD	Tumor cells	23,417	2,842	12.136
LUAD	Alveolar cell type 2	4,307	1,193	27.699
LUAD	transitional club/AT2	13,610	2,184	16.047
LUAD	Ciliated	1,506	252	16.733
LUAD	Alveolar cell type 1	281	17	6.050
LUAD	Club	222	4	1.802
LUAD	other	26	5	19.231
LUSC	Tumor cells	30,713	276	0.899
LUSC	Alveolar cell type 2	426	80	18.779
LUSC	transitional club/AT2	876	37	4.224
LUSC	Ciliated	866	125	14.434
LUSC	Alveolar cell type 1	39	2	5.128
LUSC	Club	262	0	0.000
LUSC	other	4	0	0.000

Note: Epithelial subtypes were based on the atlas-provided cell_type_major annotation. The atlas-provided "other" category is shown without further reclassification. *CDKL2*-positive cells were defined by detectable counts (>0).

Supplementary Table S11. Donor-level and within-study analyses of malignant-cell *CDKL2* detection and expression.

Analysis	LUAD	LUSC	Statistical comparison
Integrated LuCA atlas, ≥50 malignant cells/donor	n = 53; median 8.48% (IQR 1.96–20.2%)	n = 25; median 0.706% (IQR 0.0608–1.35%)	Descriptive (no inferential test)
Wu_Zhou_2021, ≥50 malignant cells/donor	n = 17; median 6.59% (IQR 1.70–11.9%)	n = 17; median 0.706% (IQR 0.0608–1.35%)	Wilcoxon W = 238; p = 0.0013
Wu_Zhou_2021 malignant-cell pseudobulk	n = 17 donors	n = 17 donors	log2FC = 3.66 (95% CI 2.49–4.83); $p = 7.87 \times 10^{-10}$; FDR-adjusted $p = 5.41 \times 10^{-8}$

Note: Donor-level analyses were restricted to samples containing at least 50 annotated malignant cells to reduce instability in detection estimates based on very small cell numbers. The integrated LuCA comparison was considered descriptive because the atlas combines multiple source studies. A formal within-study comparison was therefore performed in the Wu_Zhou_2021 cohort. Positive pseudobulk log2 fold change indicates higher *CDKL2* expression in LUAD than in LUSC. The reported pseudobulk FDR-adjusted p-value is 5.411894×10^{-8} (rounded to 5.41×10^{-8}).

Supplementary Table S12. Sensitivity analysis of donor-level malignant-cell *CDKL2* detection using different minimum malignant-cell thresholds.

Minimum malignant cells/donor	Histology	n donors	Median *CDKL2*-positive malignant cells (%)	IQR (%)
≥50	LUAD	53	8.48	1.96–20.2
≥50	LUSC	25	0.706	0.0608–1.35
≥100	LUAD	40	7.76	1.63–18.8
≥100	LUSC	21	0.706	0.115–1.17
≥200	LUAD	30	7.76	1.54–20.5
≥200	LUSC	18	0.850	0.149–1.34

Note: Donor-level analyses were repeated using minimum thresholds of 50, 100, and 200 annotated malignant cells per donor. The higher malignant-cell *CDKL2* detection observed in LUAD relative to LUSC remained directionally consistent across all thresholds. No inferential p-values were calculated for these integrated-atlas threshold comparisons because the atlas combines multiple source studies.

Abbreviations: cDC, conventional dendritic cell; pDC, plasmacytoid dendritic cell; NK, natural killer cell.

Supplementary Table S13A. Schoenfeld residual tests for the primary progression-free interval Cox model.

Variable	χ^2	df	p-value
CDKL2 expression	6.636	1	0.010
Histologic subtype	0.106	1	0.745
Age	2.797	1	0.094
Sex	1.739	1	0.187
Pathologic stage	17.187	2	< 0.001
Smoking status	0.283	1	0.595
CDKL2 × histologic subtype interaction	0.954	1	0.329
Global test	25.968	8	0.001

Note: The primary multivariable PFI Cox model included 943 participants and 323 PFI events. The proportional-hazards assumption was evaluated using Schoenfeld residuals. Evidence of nonproportionality was observed for *CDKL2*

expression (p = 0.010) and pathologic stage (p < 0.001), with a significant global test (p = 0.001). No evidence of nonproportionality was observed for the *CDKL2* × histologic subtype interaction (p = 0.329). PFI, progression-free interval.

Supplementary Table S13B. Stage-stratified Cox sensitivity analysis for progression-free interval.

Variable	Adjusted HR	95% CI	p-value
CDKL2 expression per 1 pooled-SD increase (LUAD)	0.752	0.632–0.896	0.001
Histologic subtype: LUSC vs. LUAD	0.551	0.412–0.738	< 0.001
CDKL2 expression per 1 pooled-SD increase (LUSC)	1.110	0.868–1.420	0.406
CDKL2 × histologic subtype interaction	1.475	1.094–1.989	0.011
Age per year	1.008	0.996–1.021	0.178
Sex: male vs. female	1.077	0.852–1.361	0.537
Smoking: ever vs. never	0.857	0.590–1.244	0.416

Note: This sensitivity model included 943 participants and 323 PFI events and was stratified by pathologic stage because stage showed marked evidence of nonproportionality in the primary PFI model. In the stage-stratified model, the term-specific Schoenfeld test for CDKL2 expression was no longer statistically significant (p = 0.056), and no evidence of nonproportionality was observed for the CDKL2 × histologic subtype interaction (p = 0.744). The global proportional-hazards test was also nonsignificant (p = 0.216). LUAD was the reference histologic subtype. *CDKL2* expression was log2-transformed and standardized across the pooled complete-case cohort; HRs represent the effect per 1 pooled-SD increase. The histologic subtype coefficient represents the LUSC-versus-LUAD comparison at *CDKL2* z = 0. PFI, progression-free interval; SD, standard deviation; HR, hazard ratio; CI, confidence interval.

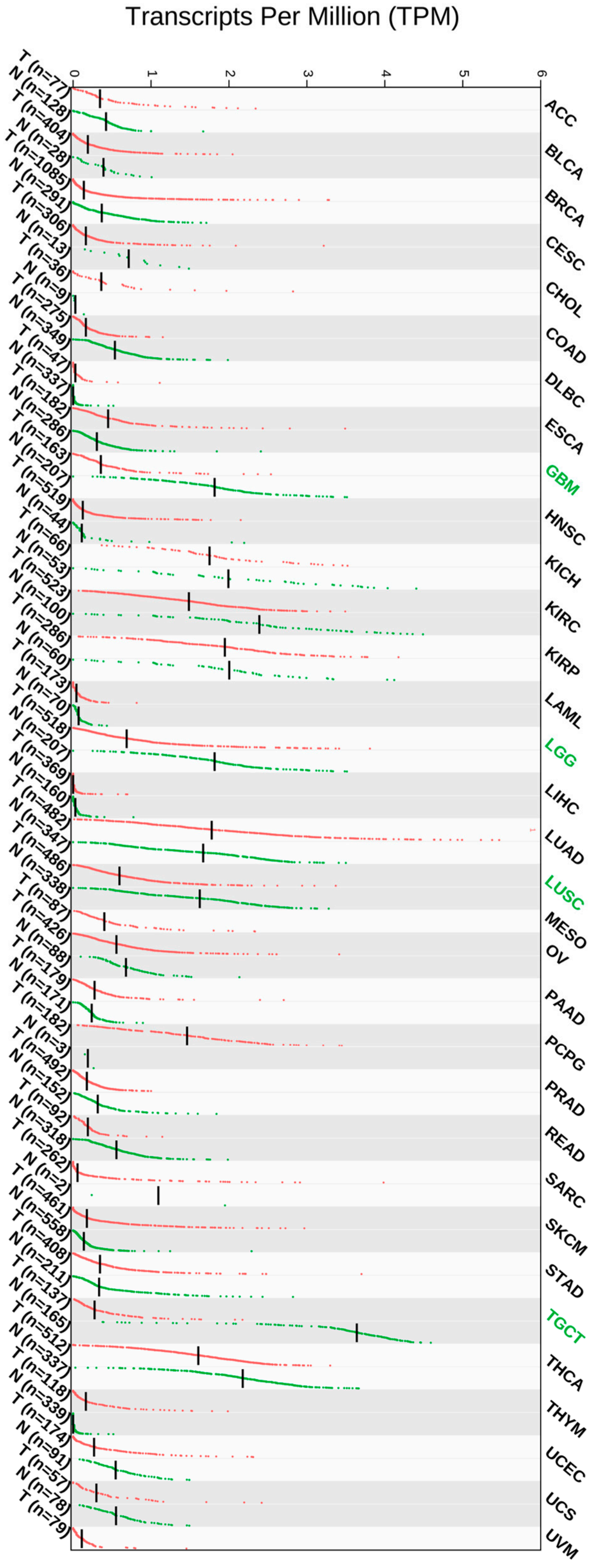

Supplementary Figure S1. Pan-cancer *CDKL2* expression in tumor (T) and reference normal (N) tissues using GEPIA2. Tumor samples are from TCGA; normal references may include TCGA and GTEx tissues depending on cancer type. This analysis is shown as complementary pan-cancer context.

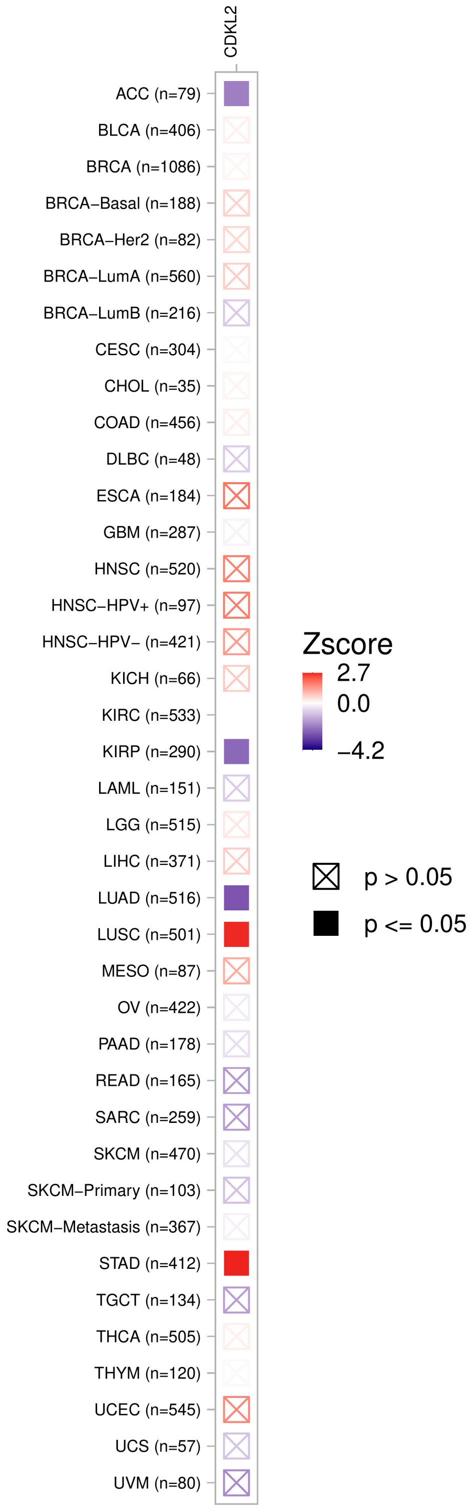

Supplementary Figure S2. Pan-cancer Cox proportional-hazards analysis of *CDKL2* expression using TIMER2.0. The prognostic association between continuous *CDKL2* expression and OS was evaluated across TCGA cancer types using the TIMER2.0 framework with adjustment for age, gender, tumor stage, and tumor purity. Red indicates increased mortality risk, blue indicates decreased mortality risk, and crossed boxes indicate non-significant associations. In LUAD, *CDKL2* expression was associated with decreased mortality risk, whereas in LUSC, *CDKL2* expression was associated with increased mortality risk. Because TIMER2.0 is largely based on TCGA samples, this analysis was interpreted as complementary sensitivity evidence rather than independent validation.

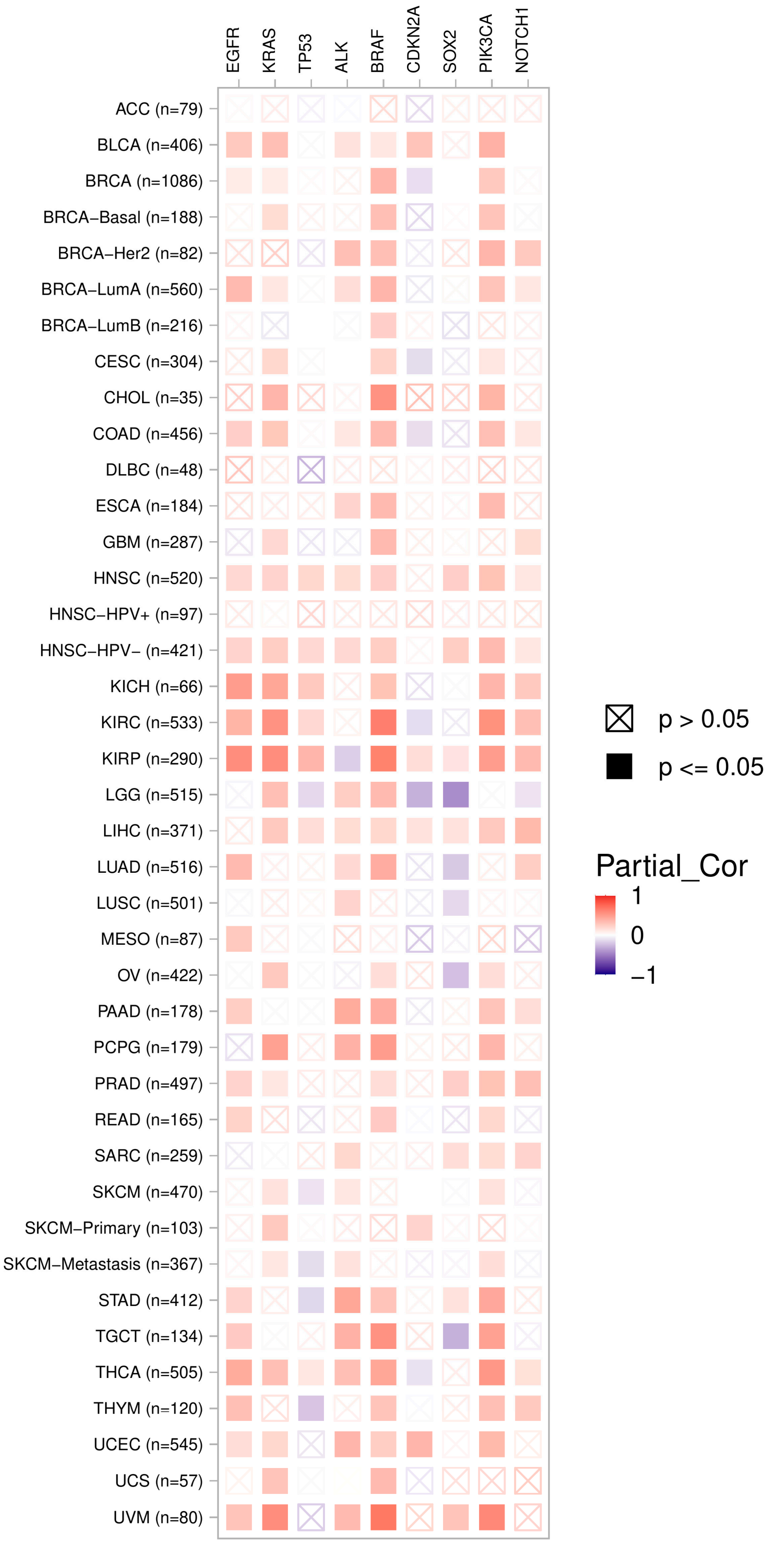

Supplementary Figure S3: Pan-cancer tumor-purity-adjusted mRNA expression correlations between *CDKL2* and selected lung cancer-related genes using TIMER2.0. Partial Spearman correlations between *CDKL2* expression and selected transcripts were evaluated across TCGA cancer types with adjustment for tumor purity. Red indicates positive correlation, blue indicates negative correlation, and crossed boxes indicate non-significant associations. These analyses represent transcript-to-transcript associations and do not assess mutation, copy-number alteration, or pathway-activation status.

A

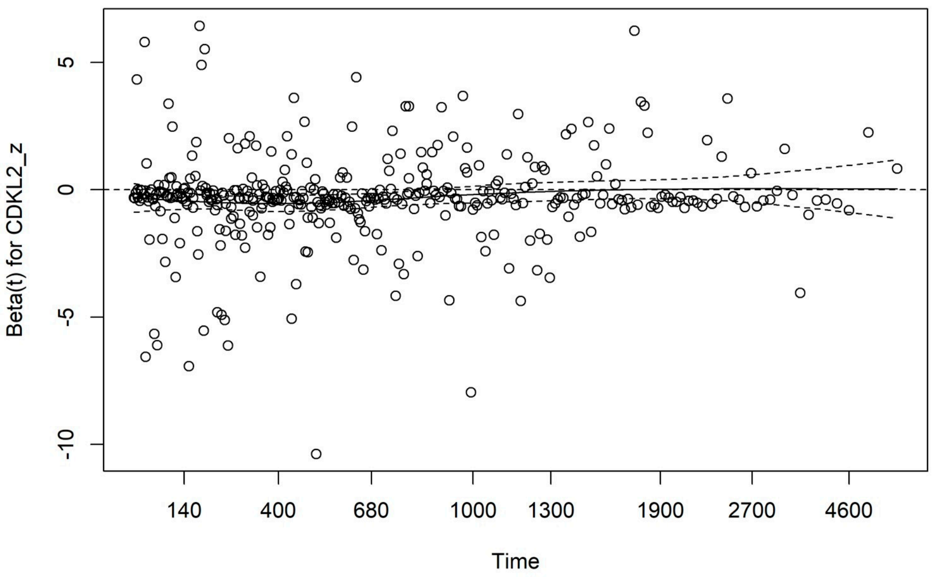

B

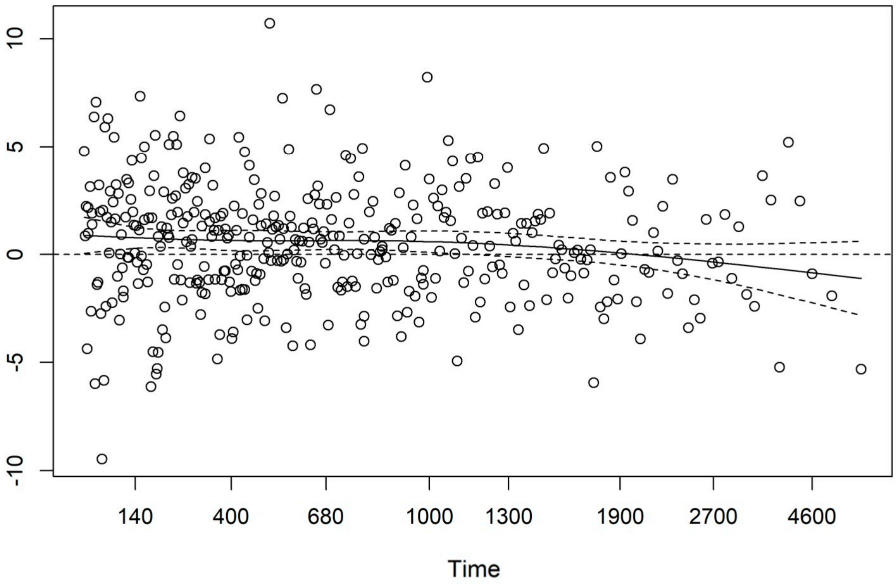

Supplementary Figure S4. Schoenfeld residual diagnostics for the primary TCGA Cox proportional-hazards model. Schoenfeld residual-based diagnostics were used to evaluate the proportional-hazards assumption for the continuous *CDKL2* effect and the *CDKL2* × histology interaction in the pooled TCGA LUAD/LUSC model. (A) Diagnostic plot for standardized continuous *CDKL2* expression; no statistically significant evidence of nonproportionality was observed (p = 0.059). (B) Diagnostic plot for the *CDKL2* × histology interaction; there was no evidence of violation of the proportional-hazards assumption (p = 0.962). Although the overall proportional-hazards test for the primary model was significant (global p = 0.012), this was attributable primarily to sex and pathologic stage rather than the *CDKL2* × histology interaction. LUAD, lung adenocarcinoma; LUSC, lung squamous cell carcinoma; TCGA, The Cancer Genome Atlas.

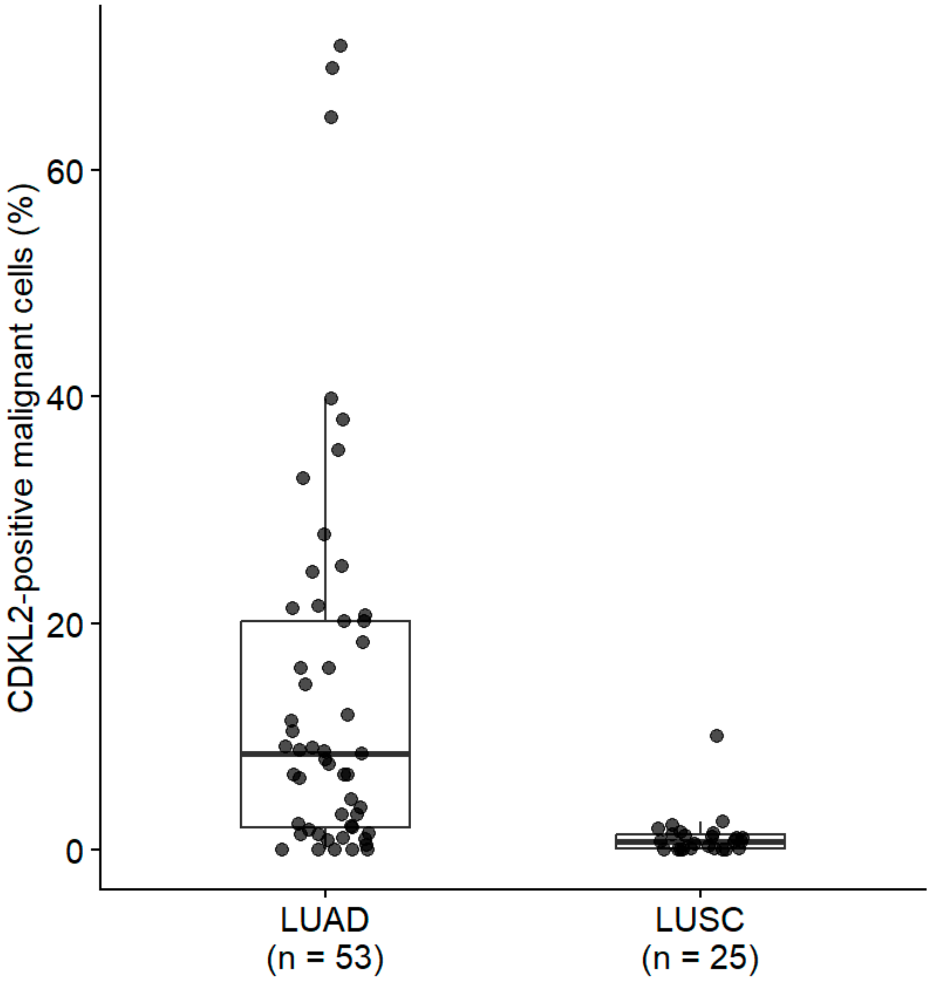

Supplementary Figure S5. Donor-level detection of *CDKL2* in annotated malignant cells from primary LUAD and LUSC tumors in the LuCA core atlas. Each point represents one donor with at least 50 annotated malignant cells. Boxes indicate the median and interquartile range. Because the integrated atlas contains samples from multiple source studies, formal within-study comparison was performed separately in the Wu_Zhou_2021 cohort.

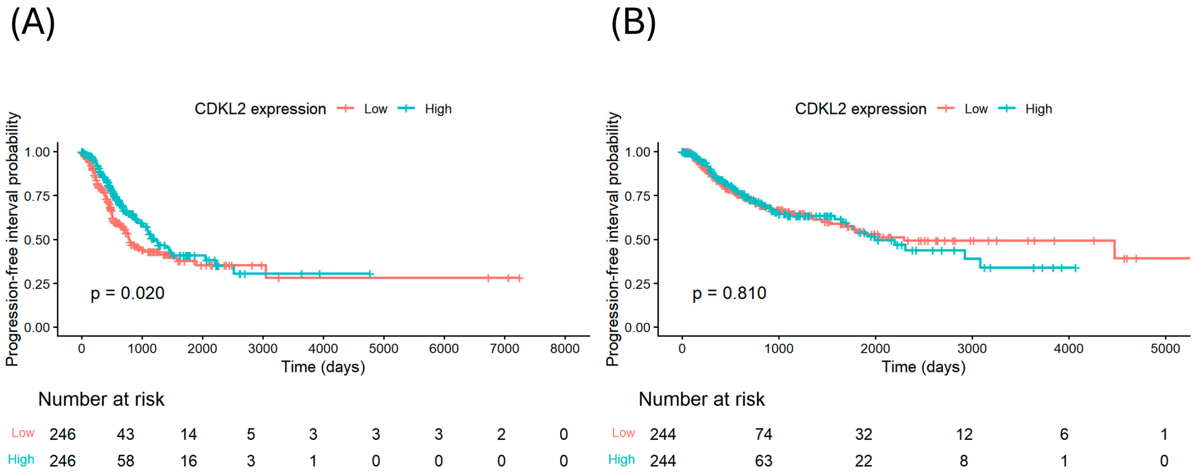

Supplementary Figure S6. Kaplan–Meier analysis of progression-free interval according to *CDKL2* expression in TCGA LUAD and LUSC. Patients

were categorized into *CDKL2*-high and *CDKL2*-low groups according to the median expression value within each histologic subtype. Kaplan–Meier curves are shown for (A) LUAD and (B) LUSC. Differences between groups were evaluated using the log-rank test. Numbers at risk are shown below each plot.
