# Supplementary material for: Prognostic Significance of CDKL2 in Non-Small Cell Lung Cancer: A Favorable Association in Lung Adenocarcinoma
Source: Medicina (Kaunas). 2026 Aug 27;62(9):1638. doi: 10.3390/medicina62091638 (PMC13609535; doi:10.3390/medicina62091638)
Supplement: Supplementary file 1 [file medicina-62-01638-s001.zip › Supplementary File S1/results/figures/Figure1_TCGA_tumor_normal_participant_level.pdf]

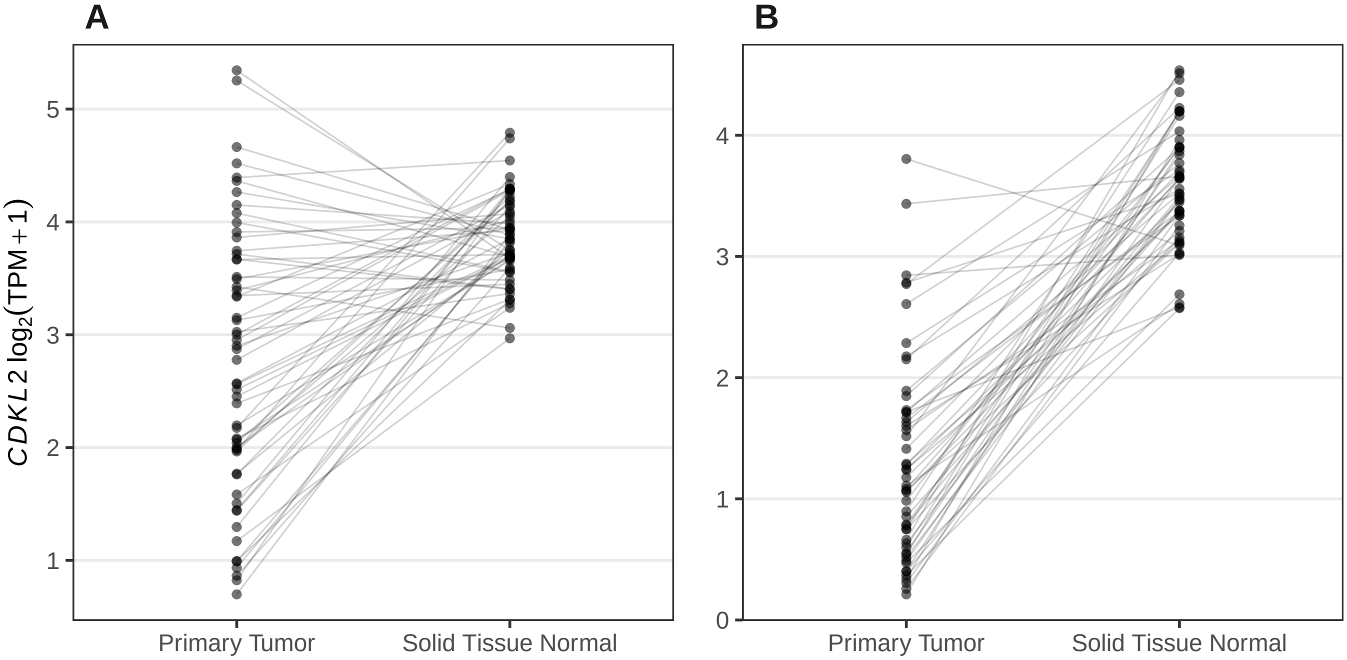

A
B
$CDKL2$ $\log_2(\mathrm{TPM}+1)$
5
4
3
2
1
0
Primary Tumor
Solid Tissue Normal
Primary Tumor
Solid Tissue Normal
