# Supplementary material for: Prognostic Significance of CDKL2 in Non-Small Cell Lung Cancer: A Favorable Association in Lung Adenocarcinoma
Source: Medicina (Kaunas). 2026 Aug 27;62(9):1638. doi: 10.3390/medicina62091638 (PMC13609535; doi:10.3390/medicina62091638)
Supplement: Supplementary file 1 [file medicina-62-01638-s001.zip › Supplementary File S1/results/figures/Figure2B_TCGA_OS_KM_LUSC.pdf]

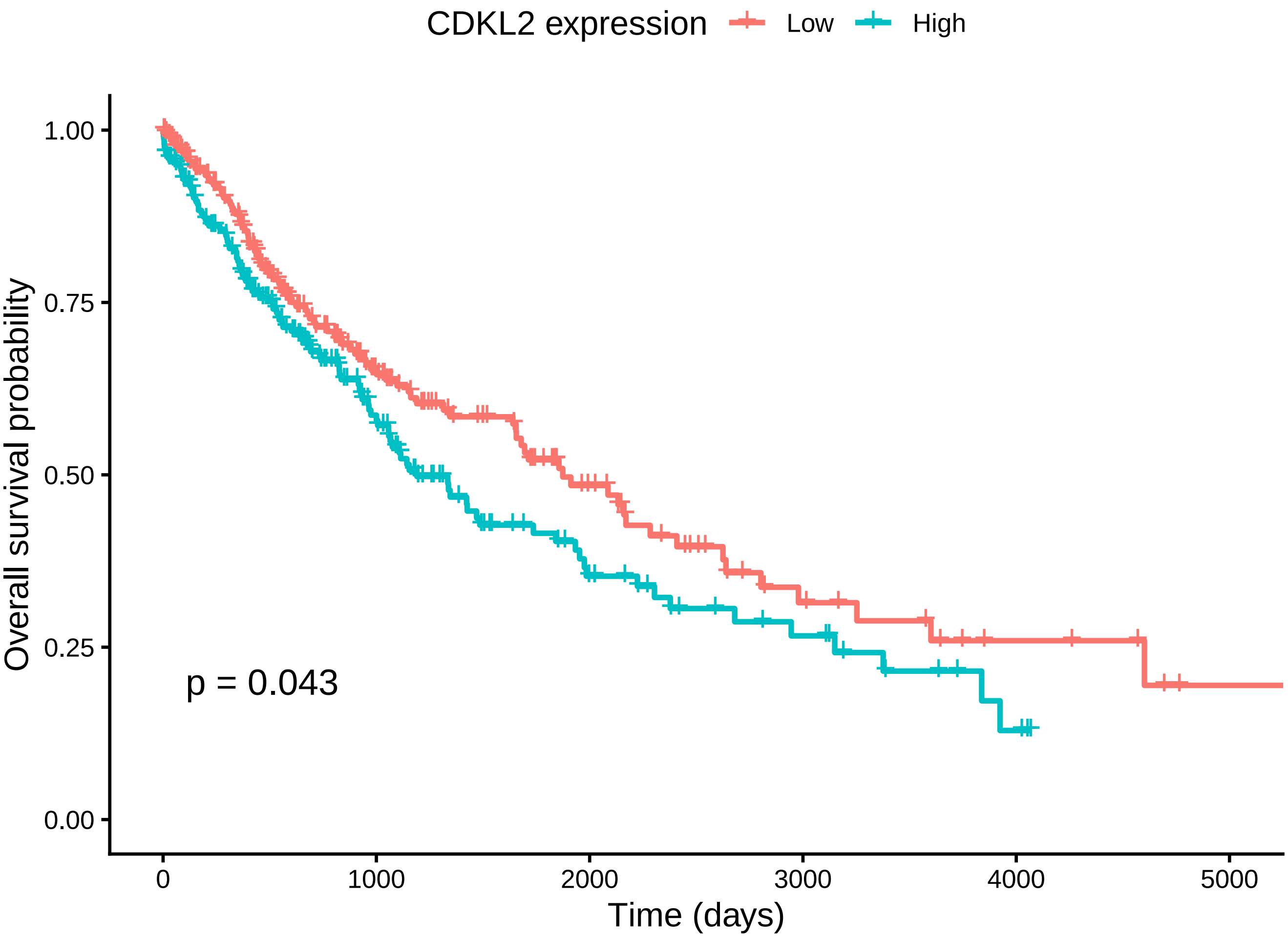

Number at risk

Low	244	84	37	14	6	1
High	244	78	27	13	3	0
