# Supplementary material for: Prognostic Significance of CDKL2 in Non-Small Cell Lung Cancer: A Favorable Association in Lung Adenocarcinoma
Source: Medicina (Kaunas). 2026 Aug 27;62(9):1638. doi: 10.3390/medicina62091638 (PMC13609535; doi:10.3390/medicina62091638)
Supplement: Supplementary file 1 [file medicina-62-01638-s001.zip › Supplementary File S1/results/figures/Figure3_KEGG_GSEA_4panel.pdf]

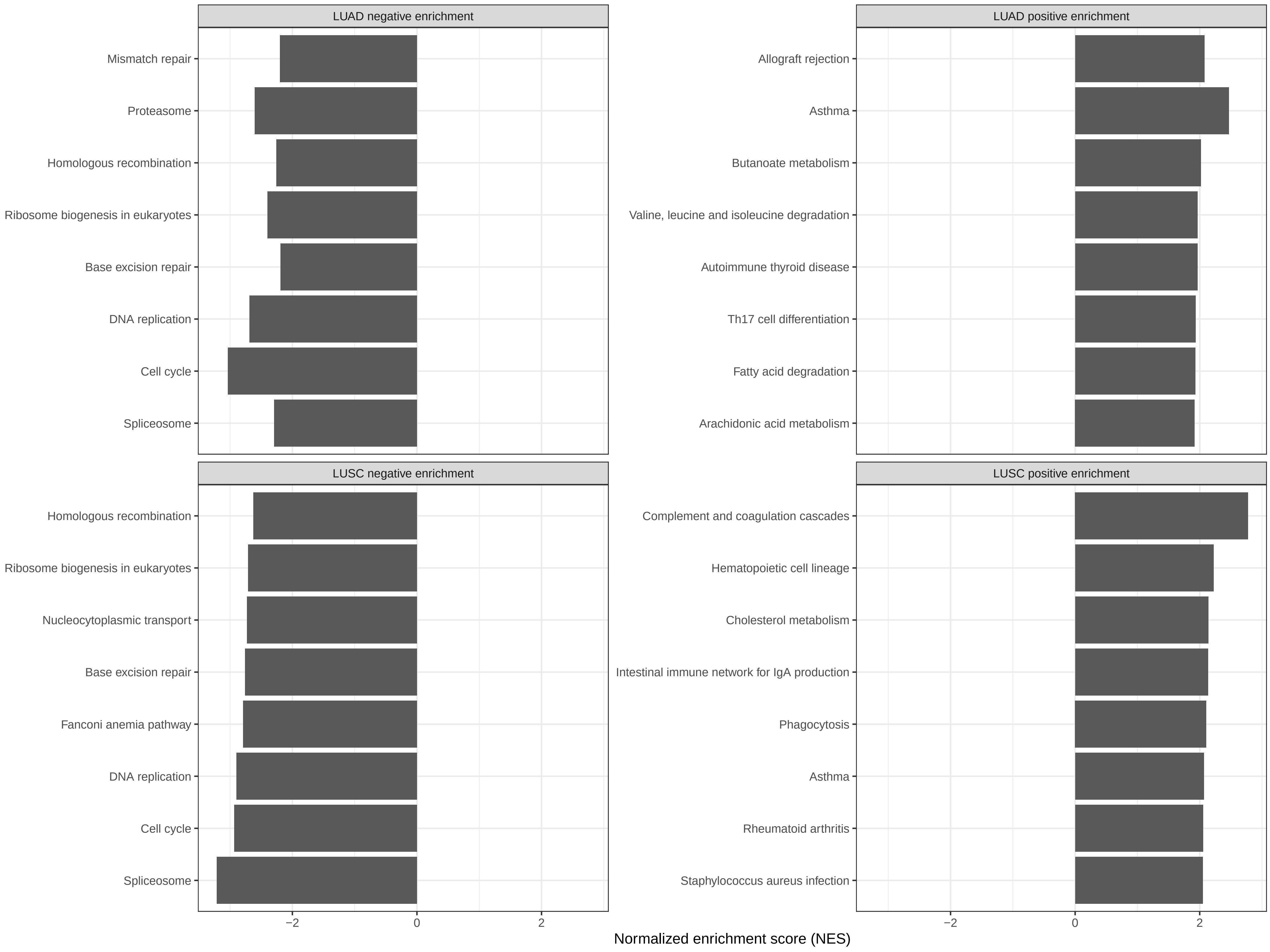

LUAD negative enrichment
Mismatch repair
Proteasome
Homologous recombination
Ribosome biogenesis in eukaryotes
Base excision repair
DNA replication
Cell cycle
Spliceosome
LUAD positive enrichment
Allograft rejection
Asthma
Butanoate metabolism
Valine, leucine and isoleucine degradation
Autoimmune thyroid disease
Th17 cell differentiation
Fatty acid degradation
Arachidonic acid metabolism
LUSC negative enrichment
Homologous recombination
Ribosome biogenesis in eukaryotes
Nucleocytoplasmic transport
Base excision repair
Fanconi anemia pathway
DNA replication
Cell cycle
Spliceosome
LUSC positive enrichment
Complement and coagulation cascades
Hematopoietic cell lineage
Cholesterol metabolism
Intestinal immune network for IgA production
Phagocytosis
Asthma
Rheumatoid arthritis
Staphylococcus aureus infection
−2
0
2
−2
0
2
Normalized enrichment score (NES)
