# Supplementary material for: Prognostic Significance of CDKL2 in Non-Small Cell Lung Cancer: A Favorable Association in Lung Adenocarcinoma
Source: Medicina (Kaunas). 2026 Aug 27;62(9):1638. doi: 10.3390/medicina62091638 (PMC13609535; doi:10.3390/medicina62091638)
Supplement: Supplementary file 1 [file medicina-62-01638-s001.zip › Supplementary File S1/results/figures/Figure4C_GSE30219_LUSC_KM.pdf]

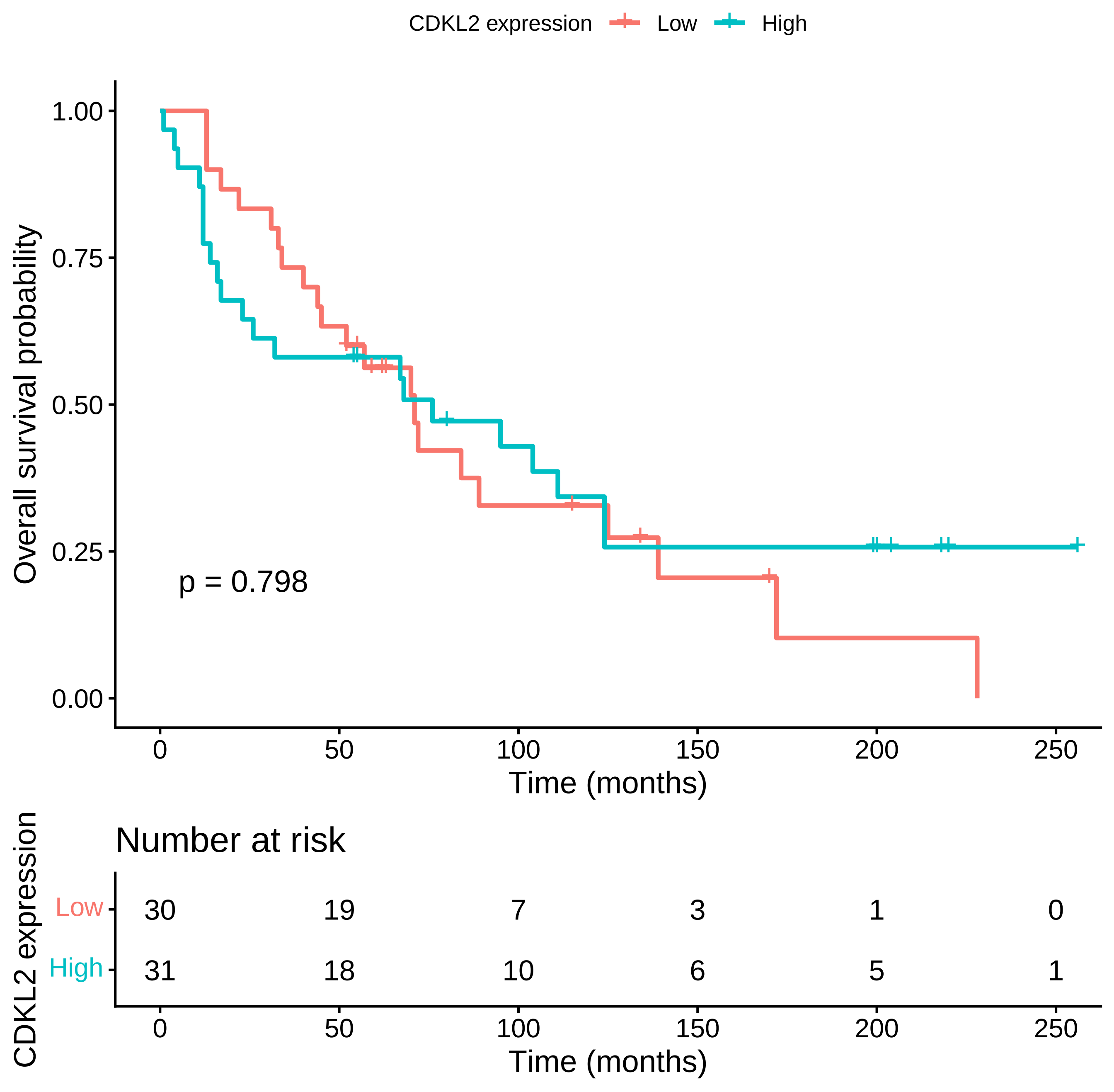
CDKL2 expression
Low
High
1.00
0.75
0.50
0.25
0.00
Overall survival probability
p = 0.798
0
50
100
150
200
250
Time (months)
Number at risk
CDKL2 expression
Low
High
30
19
7
3
1
0
31
18
10
6
5
1
0
50
100
150
200
250
Time (months)
