# Supplementary material for: Prognostic Significance of CDKL2 in Non-Small Cell Lung Cancer: A Favorable Association in Lung Adenocarcinoma
Source: Medicina (Kaunas). 2026 Aug 27;62(9):1638. doi: 10.3390/medicina62091638 (PMC13609535; doi:10.3390/medicina62091638)
Supplement: Supplementary file 1 [file medicina-62-01638-s001.zip › Supplementary File S1/results/figures/Figure4D_GSE30219_236331_at_LUSC_KM.pdf]

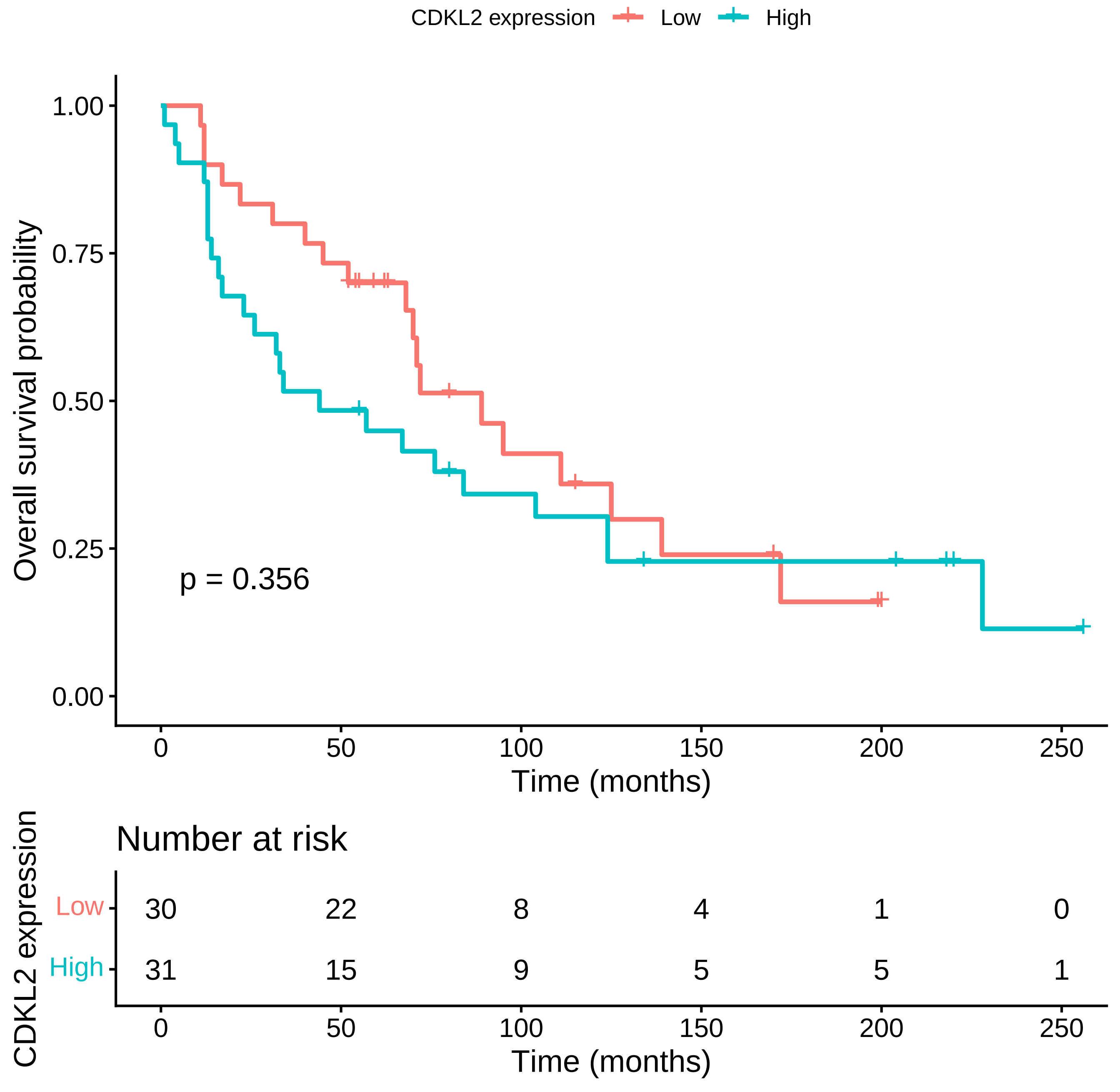

CDKL2 expression
Low
High
1.00
0.75
0.50
0.25
0.00
Overall survival probability
p = 0.356
0
50
100
150
200
250
Time (months)
Number at risk
CDKL2 expression
Low 30 22 8 4 1 0
High 31 15 9 5 5 1
0
50
100
150
200
250
Time (months)
