# Supplementary material for: Prognostic Significance of CDKL2 in Non-Small Cell Lung Cancer: A Favorable Association in Lung Adenocarcinoma
Source: Medicina (Kaunas). 2026 Aug 27;62(9):1638. doi: 10.3390/medicina62091638 (PMC13609535; doi:10.3390/medicina62091638)
Supplement: Supplementary file 1 [file medicina-62-01638-s001.zip › Supplementary File S1/results/figures/Figure4_GSE30219_parallel_two_probe_KM.pdf]

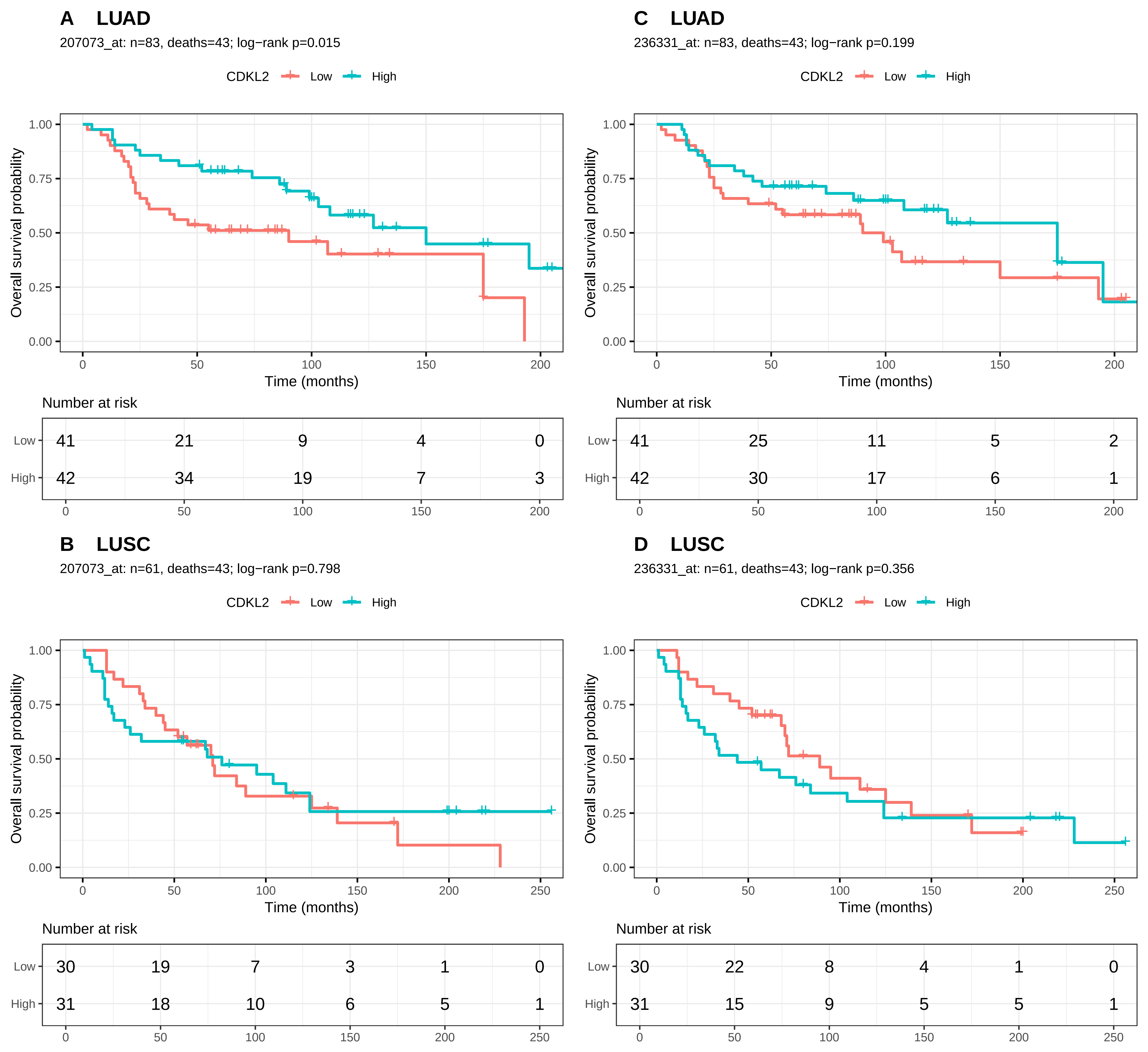
A LUAD
207073_at: n=83, deaths=43; log−rank p=0.015
CDKL2 Low High
Overall survival probability
1.00
0.75
0.50
0.25
0.00
0
50
100
150
200
Time (months)
Number at risk
Low 41 21 9 4 0
High 42 34 19 7 3
C LUAD
236331_at: n=83, deaths=43; log−rank p=0.199
CDKL2 Low High
Overall survival probability
Time (months)
Number at risk
Low 41 25 11 5 2
High 42 30 17 6 1
B LUSC
207073_at: n=61, deaths=43; log−rank p=0.798
CDKL2 Low High
Overall survival probability
250
Time (months)
Number at risk
Low 30 19 7 3 1 0
High 31 18 10 6 5 1
D LUSC
236331_at: n=61, deaths=43; log−rank p=0.356
CDKL2 Low High
Overall survival probability
Time (months)
Number at risk
Low 30 22 8 4 1 0
High 31 15 9 5 5 1
