# Supplementary material for: Prognostic Significance of CDKL2 in Non-Small Cell Lung Cancer: A Favorable Association in Lung Adenocarcinoma
Source: Medicina (Kaunas). 2026 Aug 27;62(9):1638. doi: 10.3390/medicina62091638 (PMC13609535; doi:10.3390/medicina62091638)
Supplement: Supplementary file 1 [file medicina-62-01638-s001.zip › Supplementary File S1/results/figures/Figure_S4_primary_Schoenfeld_residuals.pdf]

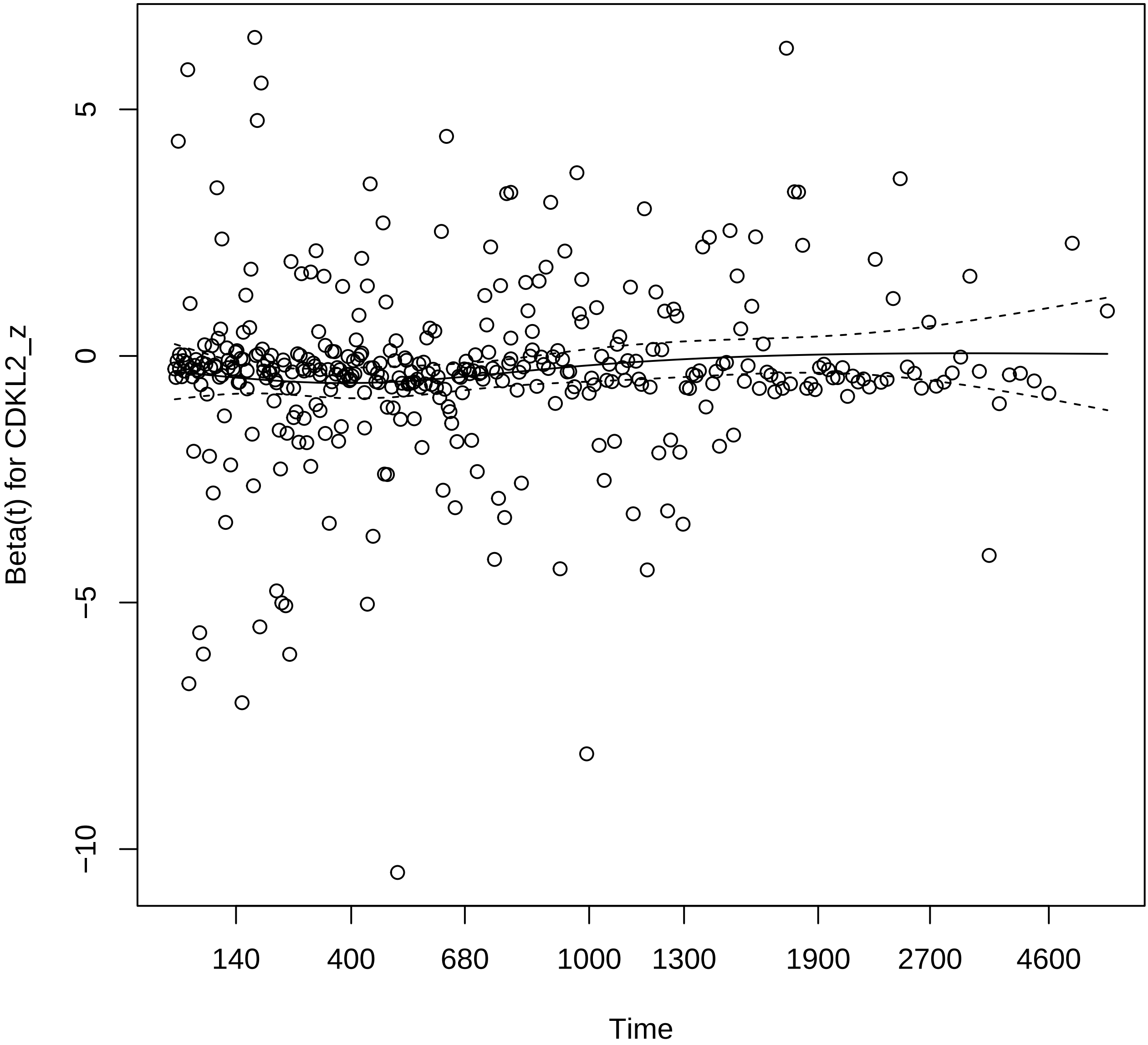

Beta(t) for CDKL2_z
5
0
−5
−10
140
400
680
1000
1300
1900
2700
4600
Time

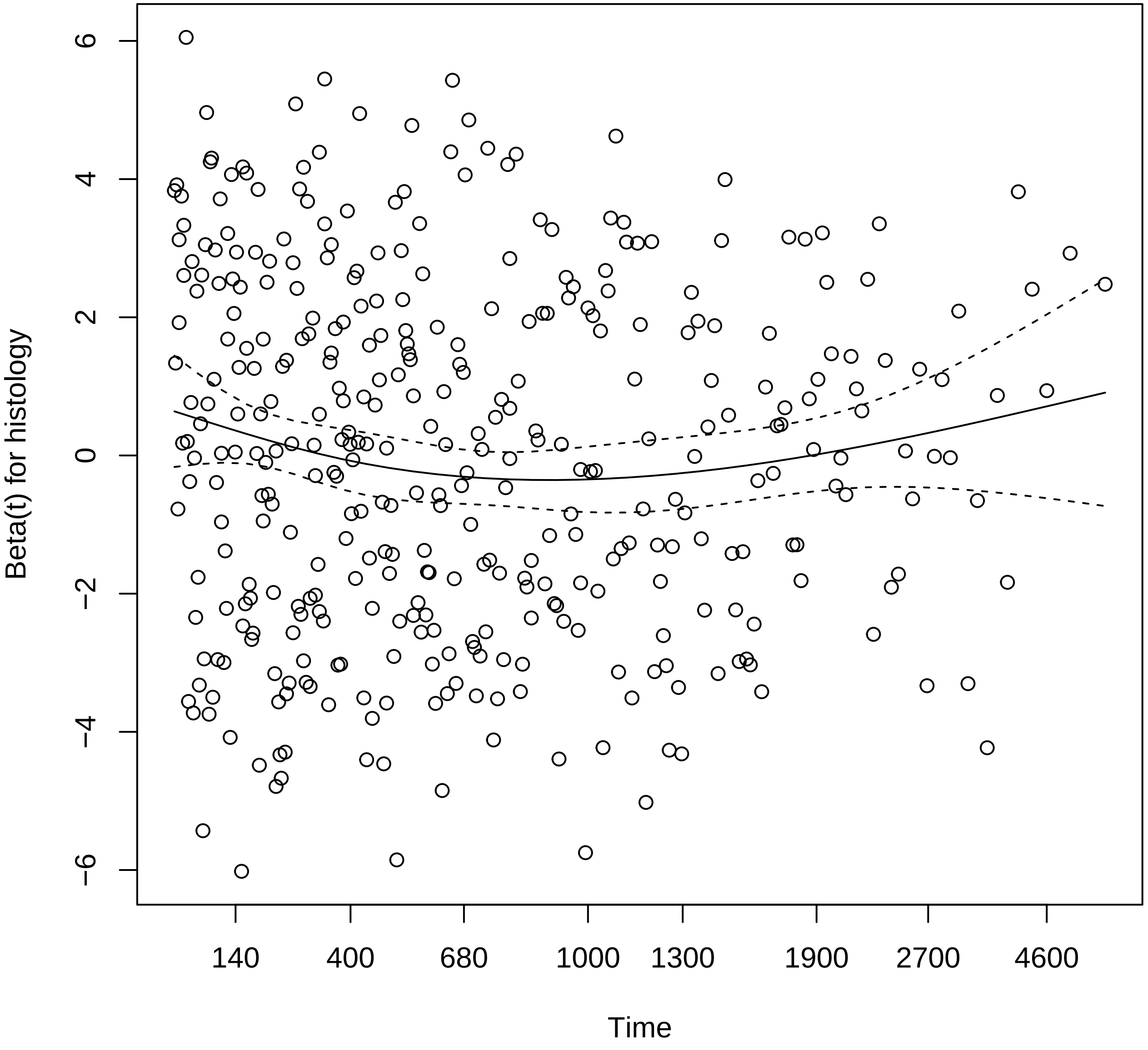

6
4
2
0
−2
−4
−6
Beta(t) for histology
140
400
680
1000
1300
1900
2700
4600
Time

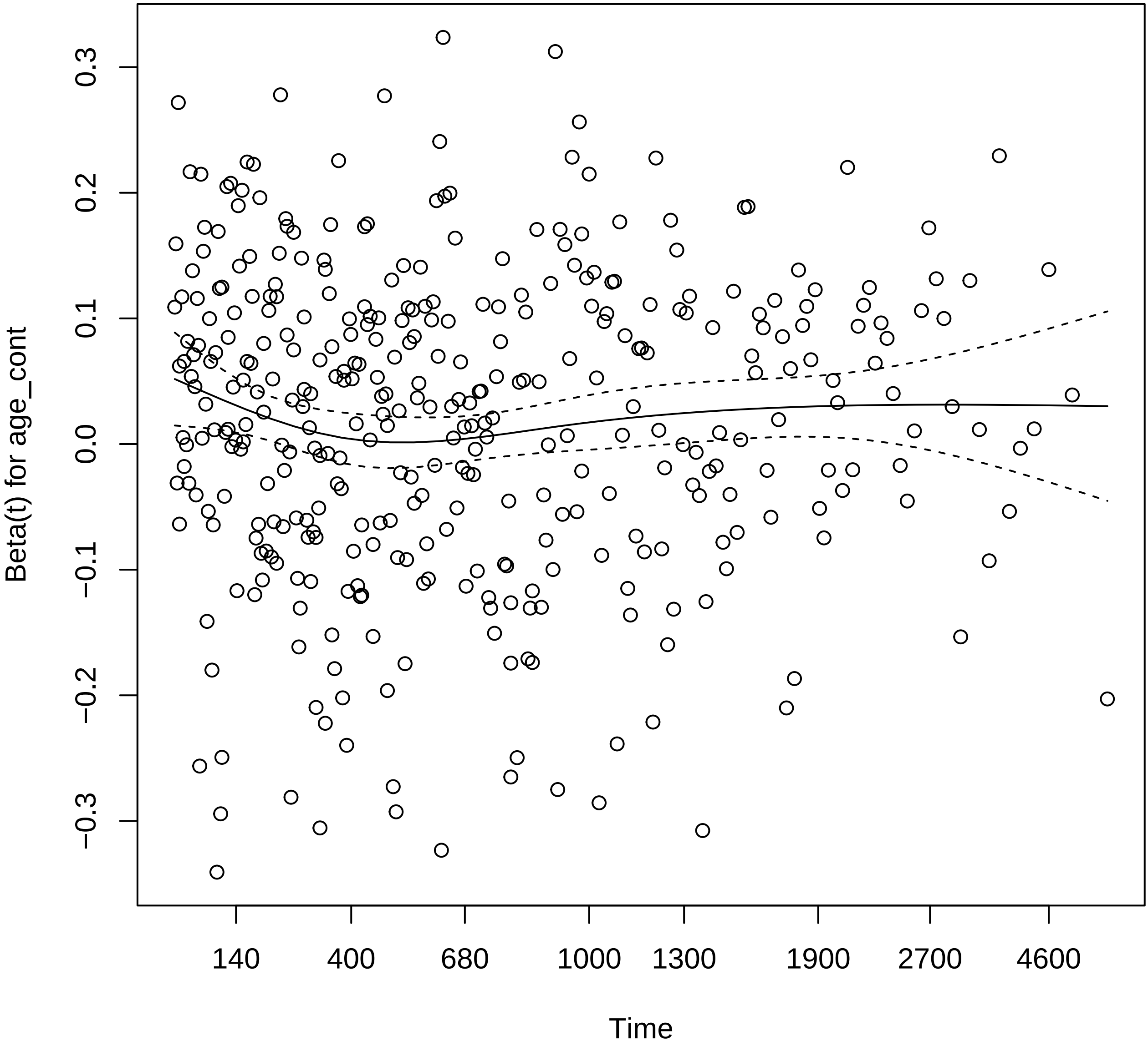

Beta(t) for age_cont
0.3
0.2
0.1
0.0
−0.1
−0.2
−0.3
140
400
680
1000
1300
1900
2700
4600
Time

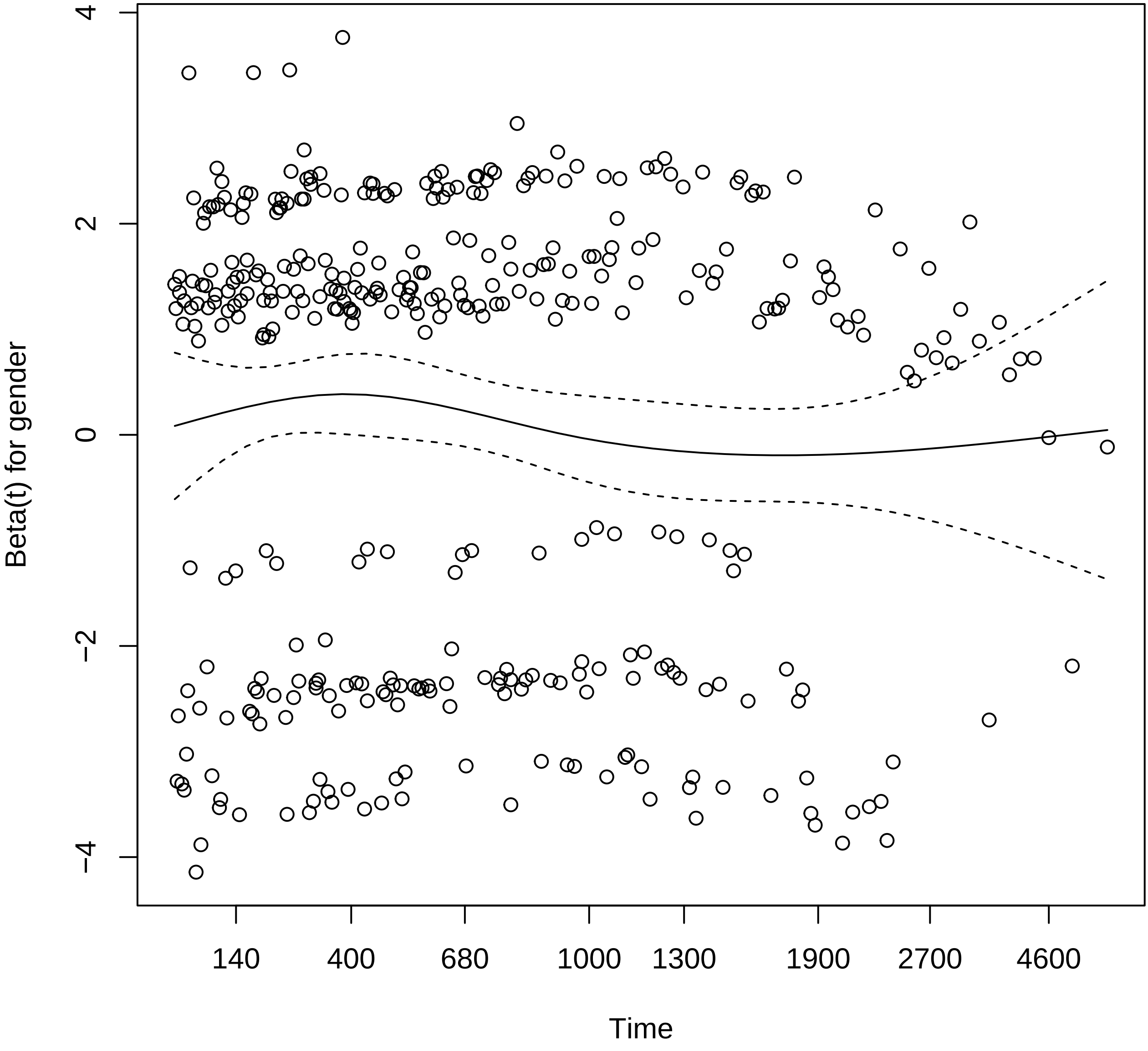

Beta(t) for gender
4
2
0
−2
−4
140
400
680
1000
1300
1900
2700
4600
Time

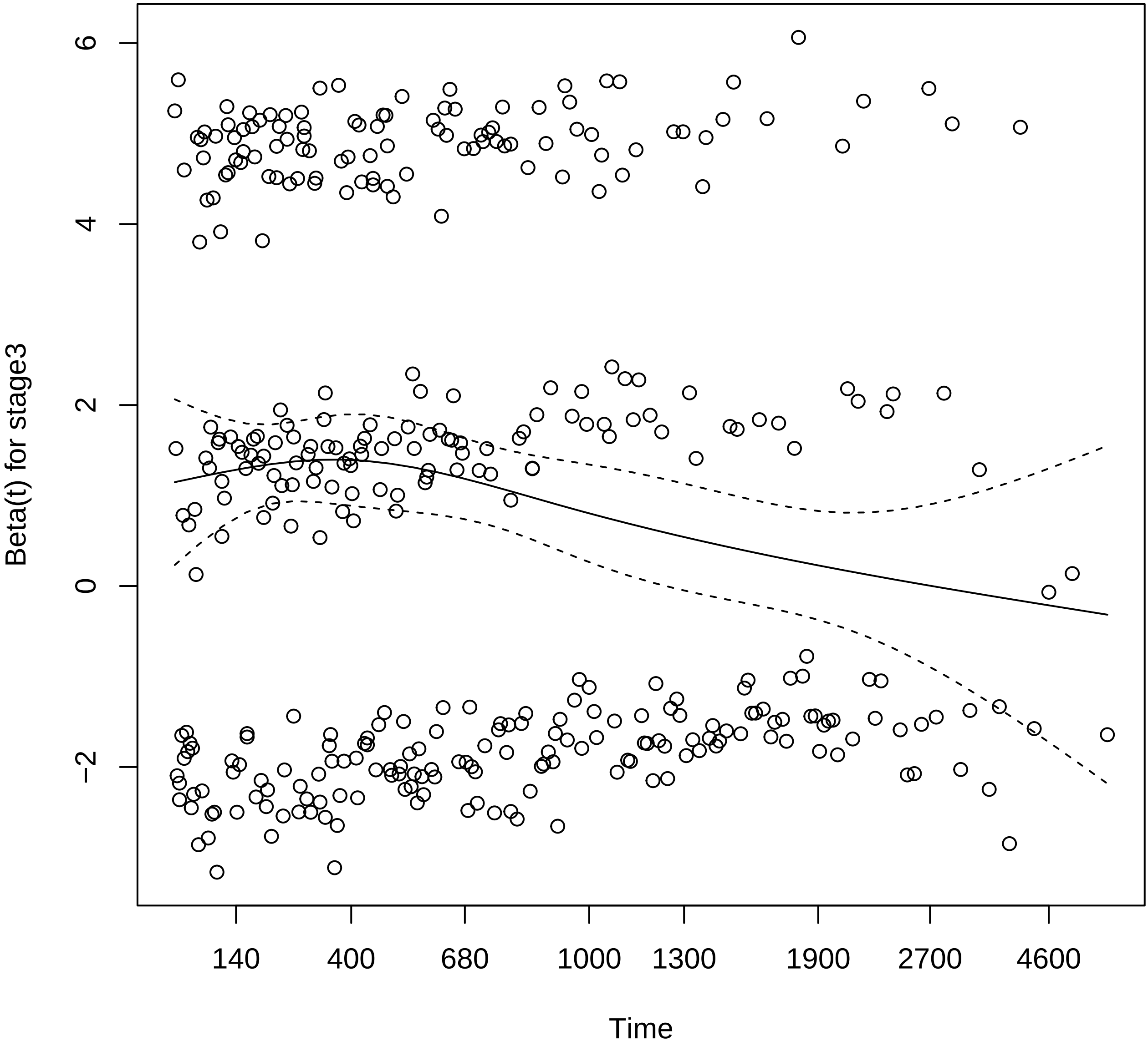

Beta(t) for stage3
6
4
2
0
−2
140
400
680
1000
1300
1900
2700
4600
Time

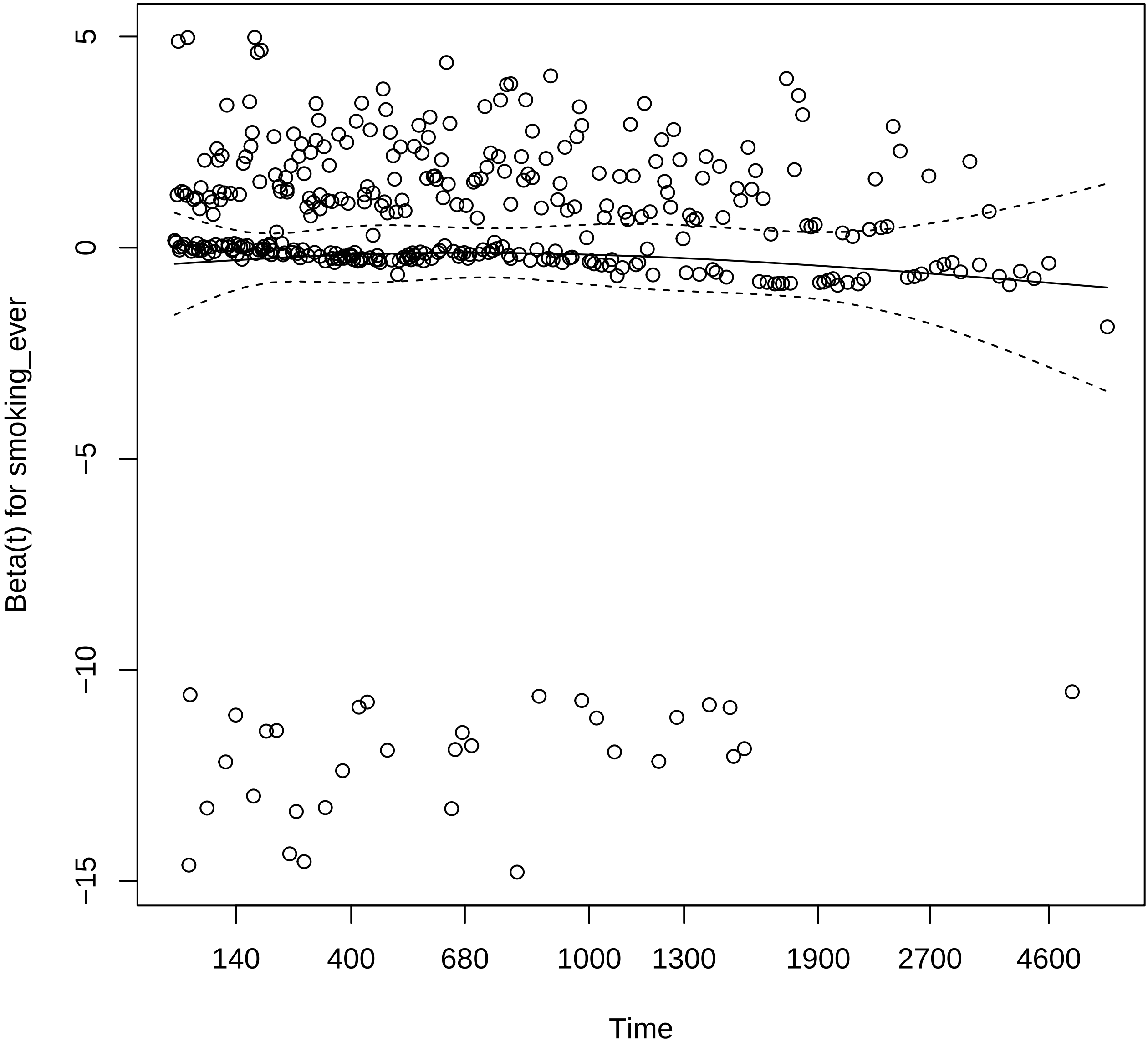

Beta(t) for smoking_ever
5
0
−5
−10
−15
140
400
680
1000
1300
1900
2700
4600
Time

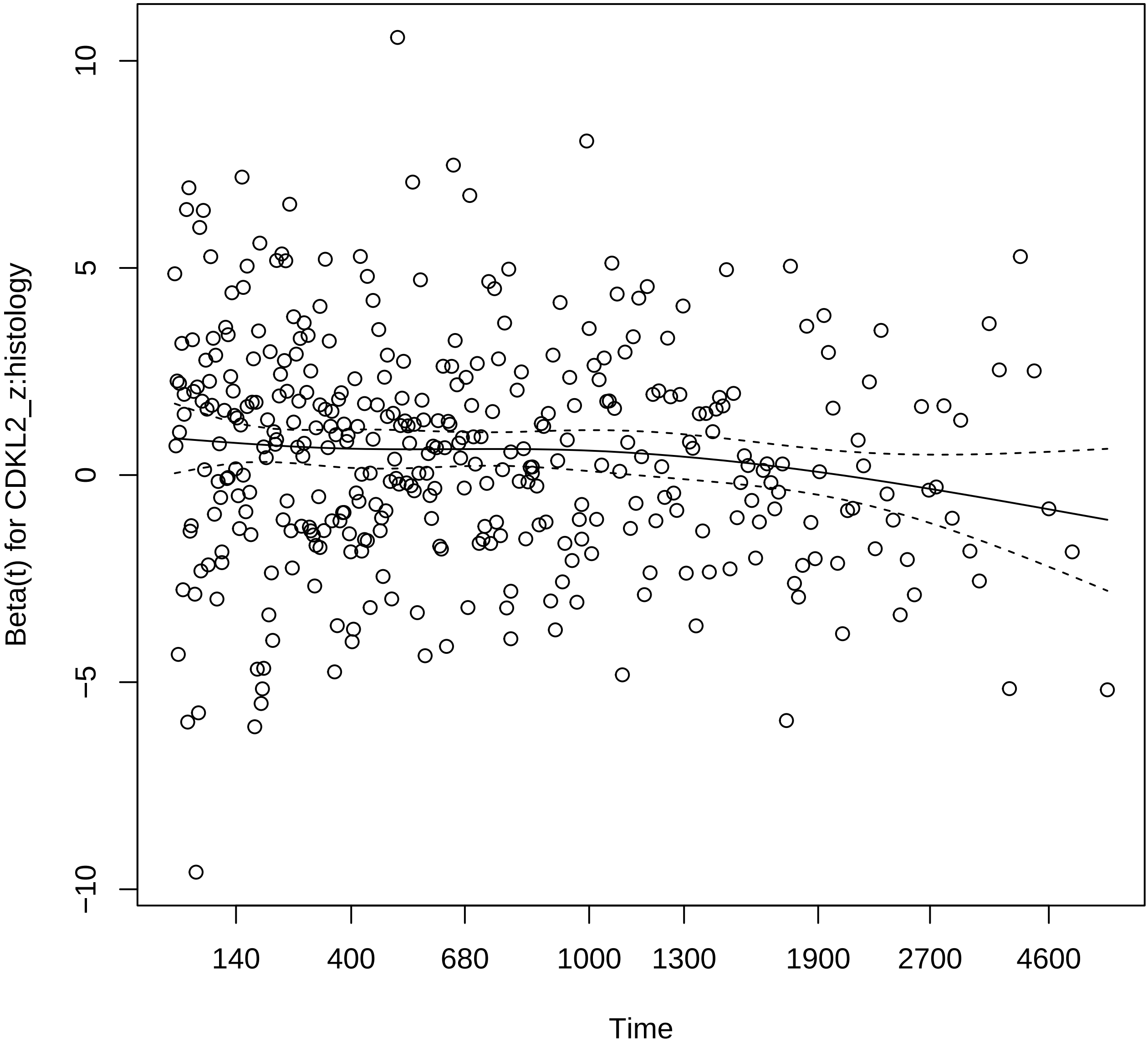

Beta(t) for CDKL2_z:histology
10
5
0
−5
−10
140
400
680
1000
1300
1900
2700
4600
Time
