# Supplementary material for: Prognostic Significance of CDKL2 in Non-Small Cell Lung Cancer: A Favorable Association in Lung Adenocarcinoma
Source: Medicina (Kaunas). 2026 Aug 27;62(9):1638. doi: 10.3390/medicina62091638 (PMC13609535; doi:10.3390/medicina62091638)
Supplement: Supplementary file 1 [file medicina-62-01638-s001.zip › Supplementary File S1/results/figures/PFI_KM_LUSC.pdf]

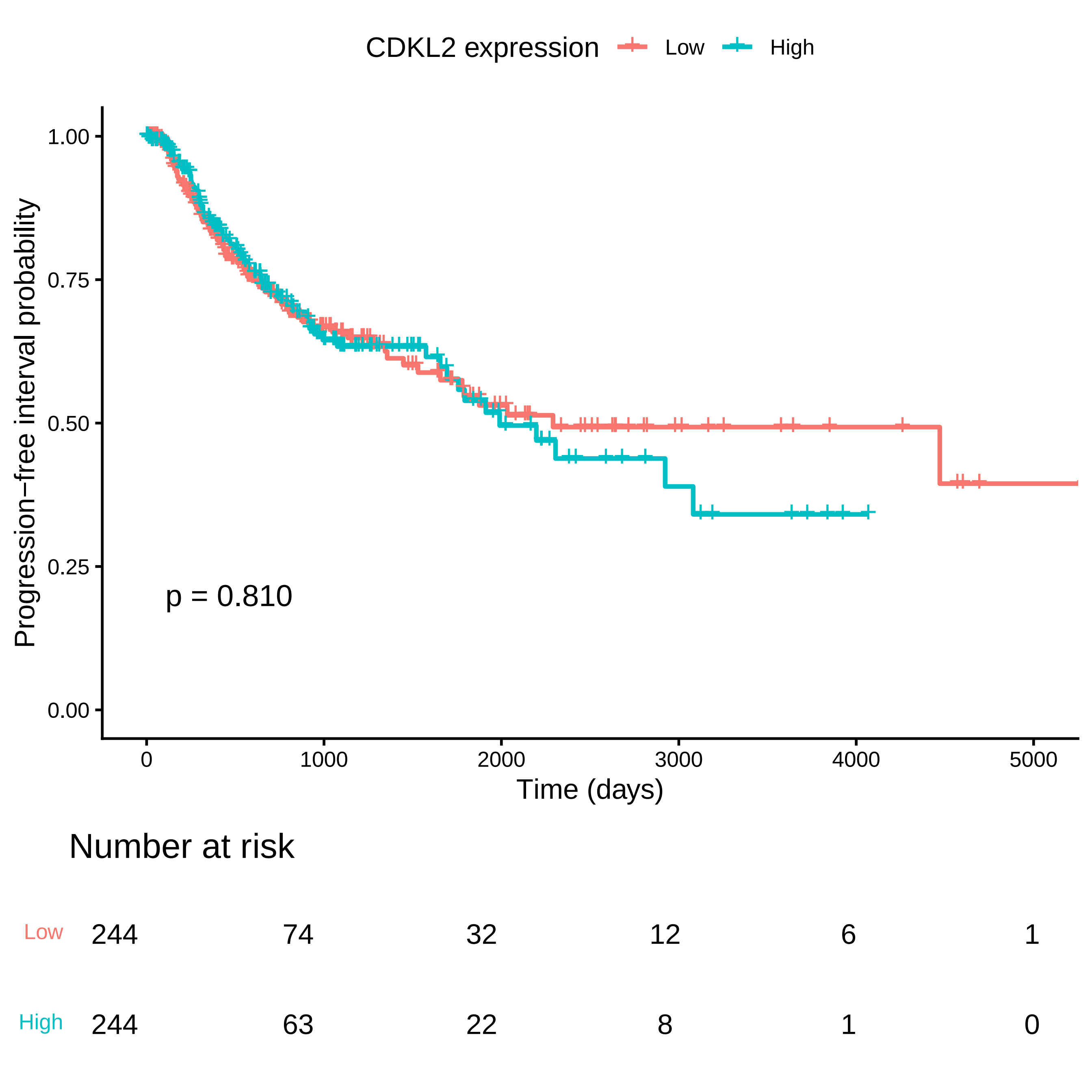
CDKL2 expression
Low
High
1.00
0.75
0.50
0.25
0.00
Progression−free interval probability
p = 0.810
0
1000
2000
3000
4000
5000
Time (days)
Number at risk
Low 244 74 32 12 6 1
High 244 63 22 8 1 0
