# Supplementary material for: Prognostic Significance of CDKL2 in Non-Small Cell Lung Cancer: A Favorable Association in Lung Adenocarcinoma
Source: Medicina (Kaunas). 2026 Aug 27;62(9):1638. doi: 10.3390/medicina62091638 (PMC13609535; doi:10.3390/medicina62091638)
Supplement: Supplementary file 1 [file medicina-62-01638-s001.zip › Supplementary File S1/results/figures/Supplementary_Figure_S5_single_cell_malignant_detection.pdf]

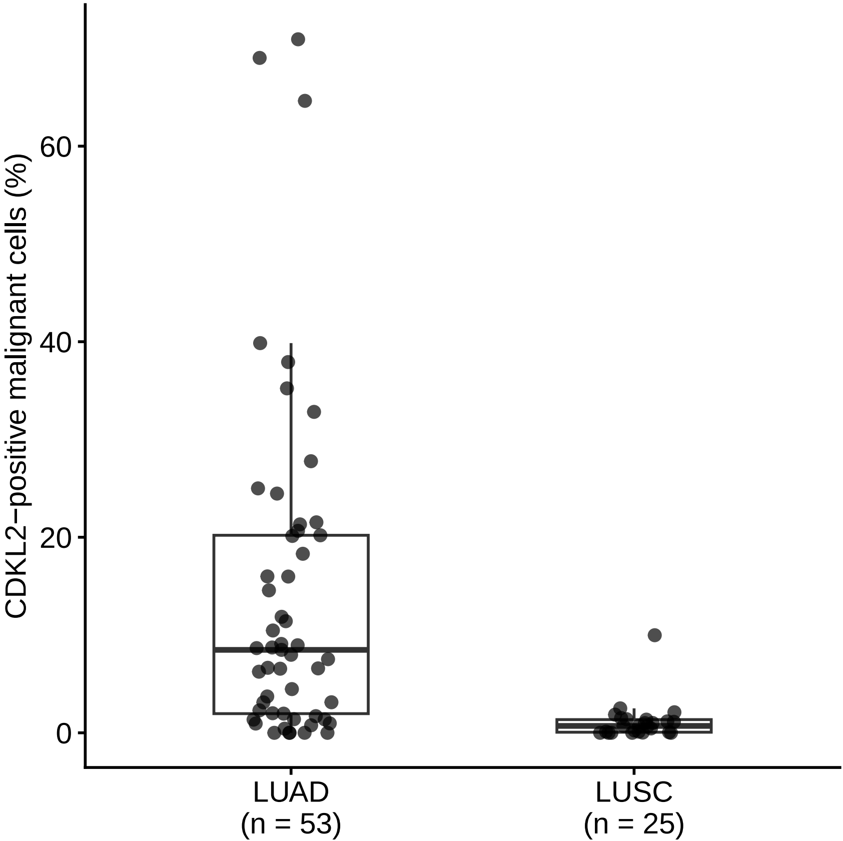

CDKL2−positive malignant cells (%)
60
40
20
0
LUAD
(n = 53)
LUSC
(n = 25)
